# Safety of COVID-19 vaccines among pregnant individuals in Quebec, Canada: a population-based retrospective cohort study from the Canadian Immunization Research Network

**DOI:** 10.1136/bmjopen-2025-106494

**Published:** 2025-12-23

**Authors:** Marilou Kiely, Ella Diendere, Isaora Dialahy, Gentiane Perrault-Sullivan, Nicholas Brousseau, Shu Qin Wei, Denis Talbot, Amélie Boutin, Caroline Quach, Sarah Jorgensen, Isabelle Boucoiran

**Affiliations:** 1 Médecine sociale et préventive, Université Laval Faculté de Médecine, Québec City, Quebec, Canada; 2 Centre de recherche du CHU de Québec-Université Laval Site CHUL, Québec City, Quebec, Canada; 3 Institut national de santé publique du Québec, Québec City, Quebec, Canada; 4 Quebec National Institute of Public Health, Quebec City, Quebec, Canada; 5 OriginElle Fertility Clinic and Women’s Health Center, Montreal, Quebec, Canada; 6 Pédiatrie, Université Laval, Québec City, Quebec, Canada; 7 Department of Epidemiology, Biostatistics and Occupational Health, University of Montreal, Montreal, Quebec, Canada; 8 Marshfield Clinic Research Institute, Marshfield, Wisconsin, USA; 9 CHU Sainte-Justine, Montreal, Quebec, Canada; 10 Université de Montréal, Montreal, Quebec, Canada

**Keywords:** Immunization Programs, INFECTIOUS DISEASES, Pregnancy, COVID-19

## Abstract

**Abstract:**

**Objective:**

To estimate the association between maternal COVID-19 vaccination during pregnancy and adverse neonatal and maternal outcomes.

**Design:**

Population-based retrospective cohort study using a hospitalisation database linked with other health administrative databases.

**Setting:**

Province of Quebec, Canada, from 1 May 2021 to 30 June 2023.

**Participants:**

All singleton pregnancies resulting in a live birth or stillbirth at ≥20 weeks of gestation, excluding those with a conception date <42 weeks before the end of the study period.

**Primary outcome measures:**

We used robust Poisson regression models to estimate adjusted risk ratios (aRRs) for chorioamnionitis, postpartum haemorrhage, caesarean delivery, preterm birth, very preterm birth, small for gestational age (SGA), maternal and neonatal admission to intensive care unit (ICU, NICU) and severe neonatal morbidity. We used a Cox regression model with a time-varying exposure variable to estimate adjusted HRs (aHRs) for stillbirth. Propensity score weighting was used to adjust for potential confounding.

**Results:**

Among 140 073 singleton pregnancies resulting in live birth or stillbirth, 61 282 individuals (43.8%) received at least one dose of messenger RNA COVID-19 vaccine during pregnancy. Vaccination during pregnancy was not associated with an increased risk of chorioamnionitis (aRR 0.99, 95% CI 0.95 to 1.04), postpartum haemorrhage (aRR 1.03, 95% CI 0.99 to 1.06), very preterm birth (aRR 1.04, 95% CI 0.89 to 1.21) and stillbirth (aHR 1.14, 95% CI 0.94 to 1.39). Vaccination during pregnancy was significantly associated with a reduced risk of caesarean delivery (aRR 0.94, 95% CI 0.92 to 0.96), maternal ICU admission (aRR 0.80, 95% CI 0.65 to 0.98), SGA (aRR 0.94, 95% CI 0.91 to 0.98), NICU admission (aRR 0.91, 95% CI 0.85 to 0.96), preterm birth (aRR 0.94, 95% CI 0.90 to 0.99) and severe neonatal morbidity (aRR 0.91, 95% CI 0.85 to 0.98).

**Conclusions:**

Our findings suggest that COVID-19 vaccination during pregnancy was not associated with an increased risk of adverse outcomes. Ongoing surveillance of the safety of maternal COVID-19 vaccination is essential as doses continue to be recommended for this group.

**STRENGTHS AND LIMITATIONS OF THIS STUDY**

- This study included a large and diverse cohort of individuals vaccinated during pregnancy at different stages of gestation.
- We used propensity score weighting to adjust for potential confounders and employed approaches that accounted for immortal time bias.
- We performed multiple subgroup and sensitivity analyses to assess the robustness of our results.
- Although we adjusted for important known confounders, residual confounding cannot be excluded.

## Introduction

 Due to important physiological and immunological changes during pregnancy, pregnant individuals are at higher risk of severe illness from SARS-CoV-2 infection compared with non-pregnant individuals of similar age.^1 2^ Indeed, pregnant individuals infected with SARS-CoV-2 are at significantly increased risk of maternal mortality, admission to intensive care unit (ICU), need for mechanical ventilation or any critical care as compared with uninfected pregnant individuals.^3 4^ Moreover, there is evidence that COVID-19 infection during pregnancy is associated with an increased risk of adverse pregnancy outcomes, such as preterm birth and stillbirth.^3 5^ Hence, preventing COVID-19 in pregnant individuals is crucial to mitigate both maternal and neonatal morbidity and mortality.

Other vaccines, such as influenza and pertussis vaccines, are recommended during pregnancy to prevent morbidity and mortality among pregnant individuals and newborns. Many countries, including Canada, have considered pregnant individuals as a priority group for COVID-19 vaccination.^6 7^ In Quebec, Canada, the COVID-19 vaccine was offered to all pregnant individuals in late April 2021, although those at higher risk of exposure or severe disease were eligible for vaccination at the start of the COVID-19 vaccination campaign in December 2020. Pregnant individuals were excluded from prelicensure trials for COVID-19 vaccines, but vaccine effectiveness among this population has been well demonstrated in observational studies.^8 9^ However, vaccination coverage among pregnant individuals was lower compared with non-pregnant individuals of reproductive age, especially among the youngest and those living in more deprived areas.^10 11^ Safety concerns are frequently cited as a reason to decline COVID-19 vaccination during pregnancy.^12^,^14^ Despite reassuring data on the safety of COVID-19 vaccines in pregnant individuals from observational studies, data from large populations, including individuals vaccinated at different trimesters of pregnancy, are still needed to better demonstrate the safety of this strategy. The availability of vaccine safety data has the potential to enhance confidence for both pregnant individuals and healthcare providers.^15^,^20^

In the context of ongoing SARS-CoV-2 circulation and the waning immunity requiring additional doses of vaccine, vigilant efforts are warranted to support timely decision-making and public health recommendations for pregnant individuals who are particularly vulnerable to more severe presentations of SARS-CoV-2 infection. In addition, as COVID-19 vaccines are still recommended during pregnancy, large-scale vaccine safety studies are needed to ensure that the benefit-risk ratio (RR) remains favourable in this population, accounting for current disease epidemiology. The purpose of this retrospective population-based cohort study was to evaluate the association between COVID-19 vaccination during pregnancy and the risk of adverse neonatal and maternal outcomes in Quebec.

## Methods

We followed the RECORD (REporting of studies Conducted using Observational Routinely-collected health Data) statement and guidelines for observational studies of COVID-19 vaccination during pregnancy.^21 22^ The study used health administrative data, and no additional information was collected from participants.

### Patients and public involvement

No patients or members of the public were involved in the conduct of this study.

### Study design, data sources and participants

We conducted a retrospective population-based cohort study among individuals who gave birth between 1 May 2021 and 30 June 2023, in hospitals in Quebec. The province of Quebec is Canada’s second most populous province, where approximately 80 000 births occur annually. The Quebec hospitalisation discharge database (ie, *Maintenance et exploitation des données pour l’étude de la clientèle hospitalière*) was used to identify the study population, using the International Statistical Classification of Diseases and Related Health Problems, 10th Revision, Canada (ICD-10-CA) and the Canadian Classification of Health Interventions (CCI) for diagnostics and procedural codes associated with vaginal or caesarean delivery. It contains data on hospital stays occurring in all public hospitals providing general and specialised care. About 97% of births in Quebec occur in a hospital.^23^ Hospital delivery records of mothers were linked with the corresponding newborn records (97% successfully linked). We deterministically linked the cohort with four other databases, using the mothers’ unique Quebec health insurance number: (1) The Provincial Laboratory Database, which captures all reverse transcription PCR (RT-PCR) confirmed SARS-CoV-2 infections. The testing strategy changed in Quebec during the study period: in January 2022, RT-PCR testing was restricted to priority groups and COVID-19 rapid antigen tests became widely used. The latter was not available in the provincial database. (2) The Quebec Vaccination Registry, which contains information on vaccine product, number of doses and vaccination dates for all vaccines administered in Quebec. All vaccine providers in Quebec are mandated to enter all administered vaccines in the registry; (3) The presence of pre-existing medical conditions was extracted from the Quebec Integrated Chronic Disease Surveillance System, which links patient-level records from provincial health system administrative databases, including hospitalisation discharge database and the physician claims database^24^; (4) Finally, maternal postal code was used to compute area-level social and material deprivation indices, which use Statistics Canada’s 2016 Census data to rank small areas according to deprivation^25^ (see online supplemental table S1 in the Supplement for a description of each data source).

All singleton pregnancies that reached at least 20 weeks’ gestation and resulted in either a live birth or stillbirth were included.^26^ To avoid fixed cohort bias, we excluded individuals who became pregnant less than 42 weeks before the end of the study.^26 27^ Pregnant individuals less than 15 years or older than 49 years of age at delivery, those vaccinated with a non-messenger RNA (mRNA) COVID-19 vaccine before delivery, those not linked to the Quebec Vaccination Registry and those with more than one pregnancy during the study period were excluded.^17 28^

### Exposure

COVID-19 vaccination campaign started in Quebec in December 2020, targeting certain pregnant individuals, such as healthcare workers or those with medical chronic conditions. Most pregnant individuals were eligible to get vaccinated as of 27 April 2021. A vaccine exposure during pregnancy was defined as the receipt of at least one dose of mRNA COVID-19 vaccine between the estimated date of conception up to 1 day before delivery, except for very preterm and preterm births, for which the exposure period was up to 31 weeks+6 days and 36 weeks+6 days gestation, respectively.^16 29 30^ The date of conception was estimated by adding 14 days to the last menstrual period date, which was derived from the gestational age at birth (validated by ultrasound) and the date of birth available in the hospitalisation records.^28 30^ Pregnant individuals who received no dose of vaccine during pregnancy (or during the specified exposure window for preterm births) were considered unexposed as well as those who received a first dose of vaccine during the postpartum period (ie, within 6 weeks after delivery). Mothers who received doses in preconception, but none during pregnancy were also considered unexposed. Vaccine exposure during pregnancy was further classified according to first trimester (from 0 to 12 weeks+6 days), second trimester (from 13 weeks to 27 weeks+6 days) and third trimester (≥28 weeks).

### Outcomes

We evaluated the following maternal complications among pregnant individuals with a live birth or stillbirth^10 17 19 22 31^: chorioamnionitis, postpartum haemorrhage, caesarean delivery, emergency caesarean delivery and ICU admission (any indication). We assessed the following neonatal outcomes among all pregnancies^10 17 22 29 31 32^: stillbirth (at ≥20 weeks of gestation), and among live births: preterm birth (≥20 weeks+0 days to 36 weeks+6 days) and very preterm birth (≥20 weeks+0 days to 31 weeks+6 days), small for gestational age (SGA) (<10th percentile of gestational age-specific and sex-specific birth weight) and neonatal ICU (NICU) admission (any indication). Preterm and very preterm births were examined overall and separately for spontaneous and provider-initiated preterm birth.^18 33^ We assessed neonatal morbidity using a composite indicator based on 15 diagnoses and 7 procedures during the birth admission. All outcomes were identified using ICD-10-CA diagnostic codes, CCI procedure codes or other data available from the maternal delivery or infant birth record, as used in previous studies.^17 34 35^ Detailed definitions are presented in online supplemental table S2 in the Supplement.

### Covariates

The following variables were considered potential confounders for the association between vaccination and maternal and neonatal outcomes: maternal age at delivery (categorised), infant sex, prepregnancy medical conditions (cardiovascular diseases, diabetes, hypertension, hypothyroidism, immunosuppression, obesity (body mass index ≥30 kg/m^2^) and respiratory diseases), healthcare worker status, tetanus-diphtheria-acellular pertussis (Tdap) vaccination during pregnancy (proxy for health behaviours), history of at least one positive SARS-CoV-2 test during pregnancy, geographical areas, month and year of conception and area-level social and material deprivation indices (see online supplemental table S2 in the Supplement for a description of each covariate).

### Statistical analysis

We compared baseline characteristics among exposed and unexposed pregnant individuals using standardised differences. An absolute standardised difference less than 0.10 was considered indicative of balance between groups.^36^ To control for potential confounding in the association between vaccination and outcomes, we computed inverse probability of treatment weights derived from the propensity score. This score represented the predicted probability, estimated from a logistic regression, of receiving at least one dose of mRNA COVID-19 vaccine during pregnancy conditional on observed baseline characteristics^36^ (see online supplemental table S3 in the Supplement). Weights were stabilised and truncated to the 1st and 99th percentiles to address the problem of large weight that can increase variability in the effect being estimated. Unadjusted and inverse probability of treatment weighted RRs and 95% CIs were computed using modified Poisson regression models with a robust, sandwich-type variance estimator to account for weight-induced dependencies.^36 37^

Since the risk of stillbirth is particularly dependent on gestational age and to address immortal time bias, we used methods that handle time-dependent exposures. Adjusted HRs (aHRs) with 95% CI were then computed using a weighted Cox-proportional-hazard regression model with gestational age in days as the time scale and follow-up time beginning at 20 weeks until the end of pregnancy or the occurrence of stillbirth.^16 18 30^ Vaccination during pregnancy was considered a time-varying exposure, with pregnant individuals contributing to both exposed and unexposed time during pregnancy. The validation of the proportional hazards assumption was based on the Schoenfeld residuals test.^38^ We also used graphical representation of the residuals as a function of time. There were 7.0% (n=9865) of missing data for material and social deprivation indices and less than 5% for infant sex (n=3802, 2.7%), prepregnancy medical condition (n=4817, 3.4%) and geographical areas (n=757, 0.5%). Missing data were not assumed to be missing at random and were handled by assigning individuals with missing data to a separate (missing) category for these variables. Statistical significance was set at 5% and the study was powered at over 80% to detect a 1% absolute increase following vaccination for rare outcomes such as stillbirth. Analyses were conducted in R (V.4.4.2) using a deidentified data set.

### Subgroup and sensitivity analyses

We performed the following subgroup and sensitivity analyses to test the robustness of our results. Data were analysed according to the number of COVID-19 vaccine doses received during pregnancy (only 1 dose, 2 doses or ≥3 doses), the trimester of pregnancy (first dose during the first trimester, first dose during the second trimester or first dose during the third trimester) and the vaccine received (BNT162b2 only, mRNA-1273 only or mixed mRNA). Since SARS-CoV-2 infection may be on the causal path between vaccination and pregnancy or neonatal outcomes, we repeated analyses excluding individuals with documented SARS-CoV-2 infection during pregnancy. As individuals who remained unvaccinated during the whole study period could differ more from the exposed group than those who got vaccinated before or after, but not during pregnancy, we limited the unexposed group to those vaccinated during the preconception or during the postpartum period, but not during pregnancy. In addition to censoring the vaccination exposure period for preterm and very preterm births to limit immortal time bias, we used a weighted Cox-proportional-hazard regression model for these outcomes using the same approach used for stillbirth. Pregnancies were followed until 36 weeks+6 days or 31 weeks+6 days. We restricted the analysis of SGA and NICU admission to term birth as these outcomes are associated with preterm birth.^18^ For SGA, we considered unexposed those vaccinated during the last 2 weeks of pregnancy as the potential effect of vaccination on fetal growth would likely require more time to have an effect.^18^ To account for the changes in COVID-19 testing strategy in Quebec during the study period, we presented results separately for births before January 2022 and for pregnant individuals with a date of conception after January 2022. Finally, given the uncertainty in the estimated date of conception, we repeated the analyses with the conception date shifted 2 weeks earlier and 2 weeks later.

## Results

**Figure 1:**
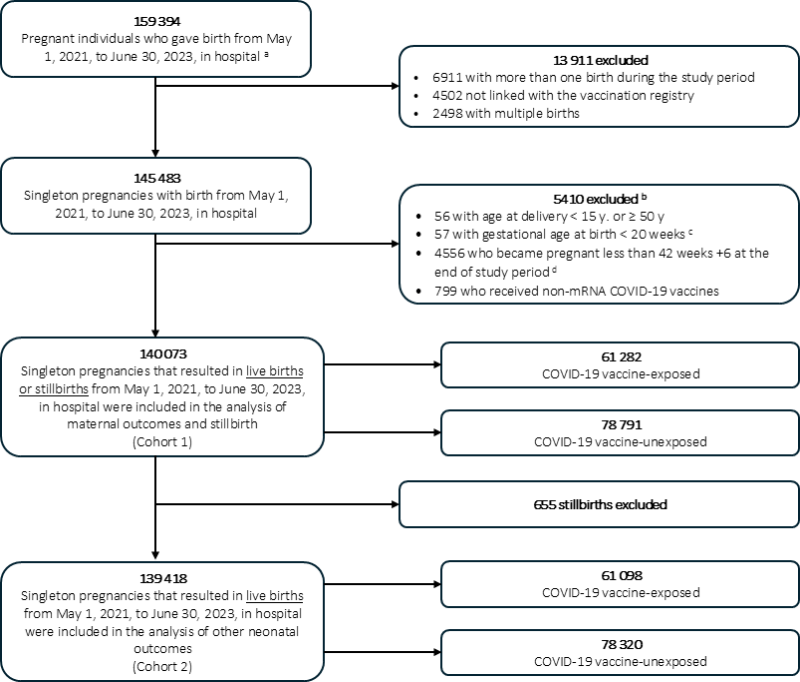
Study flowchart . ^a^Date of conception from July 2020 to September 2022. The cohort included only individuals with a unique Quebec health insurance number. ^b^Categories are not mutually exclusive. ^c^As birth at <20 weeks represents spontaneous abortion for which we do not have all full data. ^d^To avoid fixed cohort bias. mRNA, messenger RNA.

There were 159 394 pregnant individuals who gave birth in hospital in Québec from 1 May 2021 to 30 June 2023 (figure 1). After exclusions, there were 140 073 singleton pregnancies that resulted in live births or stillbirths (cohort 1), and 61 282 individuals (43.8%) received at least one dose of a mRNA COVID-19 vaccine during pregnancy. compared with those who were not vaccinated during pregnancy, those vaccinated during pregnancy were more likely to be older than 30 years of age (70.9% vs 60.5%), have a prepregnancy medical condition (26.3% vs 21.4%), be healthcare workers (17.0% vs 11.9%), have received a Tdap vaccine during pregnancy (77.1% vs 60.4%), live in the least materially deprived areas (Quintile 1: 21.5% vs 14.0%) and have conceived between October 2020 and September 2021 (table 1). After propensity score weighting, the distribution of baseline characteristics was similar between vaccinated and unvaccinated individuals, with all standardised differences less than 0.1 (table 1 and online supplemental figure S1 in the Supplement).

**Table 1:** Unweighted and weighted baseline characteristics of the total study population and by maternal COVID-19 vaccination during pregnancy

	Unweighted				Inverse probability of treatment weighted		
	Total study population N=140 073	At least 1 dose of COVID-19 vaccine during pregnancy n=61 282	No COVID-19 vaccine during pregnancy n=78 791	Standardised difference*	At least 1 dose of COVID-19 vaccine during pregnancy	No COVID-19 vaccine during pregnancy	Standardised difference*
	n (%)				%		
Maternal age group, year							
15–24	11 517 (8.2)	3243 (5.3)	8274 (10.5)	0.194	7.2	7.1	0.002
25–29	37 430 (26.7)	14 598 (23.8)	22 832 (29.0)	0.117	26.6	26.4	0.004
30–34	53 848 (38.4)	25 712 (42.0)	28 136 (35.7)	0.128	39.2	39.3	0.001
35–39	28 862 (20.6)	13 925 (22.7)	14 937 (19.0)	0.093	20.9	21.1	0.002
40–49	8416 (6.0)	3804 (6.2)	4612 (5.9)	0.015	6.1	6.1	0.002
Mean (SD)	31.5 (5.02)	32.0 (4.71)	31.0 (5.21)	0.207	31.6 (4.9)	31.6 (5.0)	0.014
Infant sex, female	66 393 (47.4)	29 173 (47.6)	37 220 (47.2)	0.007	47.5	47.6	0.002
Unknown/missing	3802 (2.7)	1449 (2.4)	2353 (3.0)				
Birth weight (≥2500 g)	133 313 (95.2)	58 622 (95.7)	74 691 (94.8)	0.040	95.6	95.1	0.024
Prepregnancy medical condition†	32 932 (23.5)	160 099 (26.3)	16 833 (21.4)	0.115	24.4	24.4	<0.001
Cardiovascular diseases	3059 (2.2)	1433 (2.3)	1626 (2.1)	0.019	2.2	2.2	<0.001
Diabetes	1187 (0.8)	550 (0.9)	637 (0.8)	0.010	0.8	0.8	<0.001
Hypertension	1578 (1.1)	735 (1.2)	843 (1.1)	0.012	1.2	1.1	<0.001
Hypothyroidism	12 314 (8.8)	6288 (10.3)	6026 (7.6)	0.092	9.2	9.3	0.001
Immunosuppression	1543 (1.1)	788 (1.3)	755 (1.0)	0.031	1.1	1.2	0.001
Obesity	10 536 (7.5)	5201 (8.5)	5335 (6.8)	0.065	8.0	7.9	0.003
Respiratory diseases	9936 (7.1)	4791 (7.8)	5145 (6.5)	0.050	7.2	7.2	<0.001
Unknown/missing‡	4817 (3.4)	635 (1.0)	4182 (5.3)				
Healthcare worker§	19 830 (14.2)	10 426 (17.0)	9404 (11.9)	0.145	14.6	14.5	0.002
Tdap¶ vaccination during pregnancy	94 828 (67.7)	47 245 (77.1)	47 583 (60.4)	0.366	69.5	69.4	0.002
Geographical areas							
Census metropolitan areas of Montreal	72 678 (51.9)	32 748 (53.4)	39 930 (50.7)	0.055	52.3	52.2	0.005
Others census metropolitan areas	28 844 (20.6)	13 589 (22.2)	15 255 (19.4)	0.069	20.7	20.7	0.001
Cities of 10 000 to 100 000 000 residents	12 769 (9.1)	5121 (8.4)	7648 (9.7)	0.047	8.9	8.8	0.001
Small cities and rural areas: less than 10 000 residents	25 025 (17.9)	9698 (15.8)	15 327 (19.5)	0.095	17.8	17.7	0.004
Unknown/missing	757 (0.5)	126 (0.2)	631 (0.8)				
History of SARS-CoV-2 infection during pregnancy	14 919 (10.7)	5925 (9.7)	8994 (11.4)	0.057	11.2	11.3	0.004
Material deprivation index							
1 (least deprived)	24 213 (17.3)	13 182 (21.5)	11 031 (14.0)	0.197	17.4	17.5	0.002
2	26 301 (18.8)	12 731 (20.8)	13 570 (17.2)	0.091	19.2	19.2	<0.001
3	26 331 (18.8)	11 710 (19.1)	14 621 (18.6)	0.014	19.2	19.2	0.001
4	26 298 (18.8)	10 406 (17.0)	15 892 (20.2)	0.082	18.9	18.8	0.001
5 (most deprived)	27 065 (19.3)	9233 (15.1)	17 832 (22.6)	0.194	18.5	18.4	0.001
Unknown/missing	9865 (7.0)	4020 (6.6)	5845 (7.4)				
Social deprivation index							
1 (least deprived)	24 511 (17.5)	11 328 (18.5)	13 183 (16.7)	0.046	17.6	17.5	0.002
2	26 172 (18.7)	11 595 (18.9)	14 577 (18.5)	0.011	18.7	18.7	<0.001
3	26 948 (19.2)	11 993 (19.6)	14 955 (19.0)	0.015	19.5	19.4	0.001
4	26 475 (18.9)	11 484 (18.7)	14 991 (19.0)	0.007	18.9	19.0	0.002
5 (most deprived)	26 102 (18.6)	10 862 (17.7)	15 240 (19.3)	0.042	18.5	18.5	<0.001
Unknown/missing	9865 (7.0)	4020 (6.6)	5845 (7.4)				
Estimated date of conception							
July 2020 to September 2020	8289 (5.9)	2526 (4.1)	5763 (7.3)	0.138	6.2	6.2	<0.001
October 2020 to December 2020	18 623 (13.3)	9741 (15.9)	8882 (11.3)	0.135	17.3	17.3	0.001
January 2021 to March 2021	17 583 (12.6)	11 243 (18.3)	6340 (8.0)	0.308	14.2	14.3	0.002
April 2021 to June 2021	16 741 (12.0)	12 142 (19.8)	4599 (5.8)	0.427	10.9	10.9	<0.001
July 2021 to September 2021	17 647 (12.6)	10 359 (16.9)	7288 (9.2)	0.229	15.5	15.5	<0.001
October 2021 to December 2021	18 435 (13.2)	7489 (12.2)	10 946 (13.9)	0.050	16.5	16.5	0.001
January 2022 to March 2022	16 393 (11.7)	3264 (5.3)	13 129 (16.7)	0.369	8.1	8.1	<0.001
April 2022 to June 2022	14 196 (10.1)	2483 (4.1)	11 713 (14.9)	0.376	6.2	6.1	0.005
July 2022 to September 2022	12 166 (8.7)	2035 (3.3)	10 131 (12.9)	0.355	5.1	5.0	0.003

* A standardised difference of less than 0.1 is considered indicative of balance between groups.

† At least one of prepregnancy medical conditions listed.

‡ Individuals not matched between MED-ÉCHO and SISMACQ could be those without health insurance coverage for at least 1 day during the year, or individuals who are not registered (such as newcomers). This could also be due to errors in the health insurance number.

§ Employees on the payroll list or members of professional associations.

¶ Tdap: tetanus-diphtheria-acellular pertussis vaccine.

MED-ÉCHO, Maintenance et exploitation des données pour l’étude de la clientèle hospitalière; SISMACQ, Le Système intégré de surveillance des maladies chroniques du Québec.

**Figure 2:**
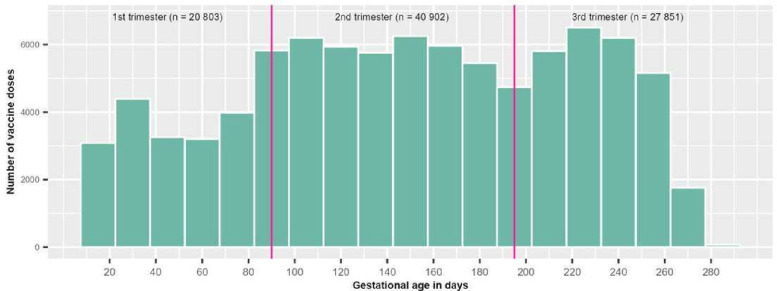
Gestational age at COVID-19 vaccination during pregnancy. Including all doses administered during pregnancy. If an individual received two doses during a trimester, both doses have been counted.

Among the 61 282 individuals vaccinated during pregnancy, 34 371 received 1 dose (65.1%) and 26 911 (43.9 %) ≥2 doses. A greater proportion of vaccinated individuals received BNT162b2 from Pfizer-BioNTech (70.4%) compared with mRNA-1273 from Moderna (26.2%), while a small proportion received mixed mRNA vaccines (3.4%) (table 2). Nearly a quarter (23.2%) of all doses administered during pregnancy were received in the first trimester (figure 2). The distribution of SARS-CoV-2 infections during pregnancy is presented in online supplemental figure S2 in the Supplement.

**Table 2:** Vaccination characteristics among pregnant individuals who received at least one dose of COVID-19 vaccine during pregnancy

Characteristics	At least one COVID-19 vaccine dose during pregnancy N=61 282, n (%)
COVID vaccinated (at least one dose) according to the trimesters of pregnancy*	
First trimester	19 442 (31.7)
Second trimester	34 854 (56.9)
Third trimester	25 769 (42.0)
Number of doses received during pregnancy	
1	34 371 (56.1)
2	25 563 (41.7)
3	1345 (2.2)
4	3 (0.0)
Dose 1 received during:†	
First trimester	19 442 (31.7)
Second trimester	29 032 (47.4)
Third trimester	12 808 (20.9)
Dose 2 received during:†	
First trimester	1361 (2.2)
Second trimester	11 818 (19.3)
Third trimester	13 732 (22.4)
No dose 2 during pregnancy	34 371 (56.1)
Dose 3 received during:†	
First trimester	0 (0.0)
Second trimester	51 (0.1)
Third trimester	1297 (2.1)
No dose 3 during pregnancy	59 934 (97.8)
Dose 4 received during:†	
First trimester	0 (0.0)
Second trimester	0 (0.0)
Third trimester	3 (0.0)
No dose 4 during pregnancy	61 279 (99.9)
Vaccine types received during pregnancy	
BNT162b2	43 137 (70.4)
mRNA-1273	16 086 (26.2)
Mixed mRNA	2059 (3.4)
Intervals between doses 1 and 2 (days)‡	
0–29	2965 (4.8)
30–55	4934 (8.1)
56+	19 012 (31.0)
Only 1 dose during pregnancy	34 371 (56.1)
Intervals between Doses 2 and 3 (days)‡	
0–119	238 (0.4)
120–179	996 (1.6)
180+	114 (0.2)
No dose 2 and/or 3 during pregnancy	59 934 (97.8)
Intervals between doses 3 and 4 (days)‡	
0–119	2 (0.0)
120+	1 (0.0)
No dose 3 and/or 4 during pregnancy	61 279 (99.9)

* Categories are not mutually exclusive as individuals could have received a dose in the first, second and third trimesters.

† Vaccination received exclusively during pregnancy. Does not take previous vaccination into account.

‡ Only for doses given during pregnancy.

mRNA, messenger RNA.

Vaccination during pregnancy was not significantly associated with an increased risk of chorioamnionitis (5.5% vs 5.7%, adjusted RR (aRR) 0.99, 95% CI 0.95 to 1.04). COVID-19 vaccination during pregnancy was not significantly associated with postpartum haemorrhage (10.8% vs 11.1%, aRR 1.03, 95% CI 0.99 to 1.06), compared with individuals not vaccinated during pregnancy. The risk of caesarean delivery was significantly lower among vaccinated individuals compared with unvaccinated individuals (25.8% vs 26.9%, aRR 0.94, 95% CI 0.92 to 0.96), as well as the risk of emergency caesarean delivery (14.9% vs 16.0%, aRR 0.95, 95% CI 0.92 to 0.98). A reduced risk was also observed for maternal ICU admission (0.31% vs 0.40%, aRR 0.80, 95% CI 0.65 to 0.98) (table 3).

**Table 3:** Association between COVID-19 vaccination during pregnancy and the risk of adverse maternal outcomes

Outcomes	N (%)			Risk ratio (95% CI)
	≥1 dose during pregnancy	No dose during pregnancy	Unadjusted	Adjusted*
Pregnancies with a live birth or stillbirth (cohort 1)	N=61 282	N=78 791		
Chorioamnionitis	3392 (5.5)	4472 (5.7)	0.98 (0.93 to 1.02)	0.99 (0.95 to 1.04)
Postpartum haemorrhage	6629 (10.8)	8757 (11.1)	0.97 (0.94 to 1.00)	1.03 (0.99 to 1.06)
Caesarean delivery	15 838 (25.8)	21 222 (26.9)	0.96 (0.94 to 0.98)	0.94 (0.92 to 0.96)
Emergency caesarean delivery	9138 (14.9)	12 629 (16.0)	0.93 (0.91 to 0.95)	0.95 (0.92 to 0.98)
Maternal ICU admission	192 (0.31)	315 (0.40)	0.78 (0.66 to 0.94)	0.80 (0.65 to 0.98)

* Adjusted for maternal age at delivery, infant sex, maternal pre-existing conditions, healthcare provider status, SARS-CoV positive test during pregnancy, social and material deprivation indexes, month and year of conception, Tdap vaccination during pregnancy and geographical areas using inverse probability of treatment weighting.

ICU, intensive care unit; Tdap, tetanus-diphtheria-acellular pertussis.

We did not find a significant association between vaccination during pregnancy and very preterm birth (0.60% vs 0.72%, aRR 1.04, 95% CI 0.89 to 1.21) or stillbirth (0.3 vs 0.4 per 10 000 pregnancy days at risk, aHR 1.14, 95% CI 0.94 to 1.39). Risks of other neonatal outcomes were lower in vaccinated individuals compared with those who were unvaccinated; these reduced risks remained significant after adjustment for SGA (9.3% vs 10.2%, aRR 0.94, 95% CI 0.91 to 0.98), NICU admission (3.8% vs 4.4%, aRR 0.91, 95% CI 0.85 to 0.96), severe neonatal morbidity (2.7% vs 3.2%, aRR 0.91, 95% CI 0.85 to 0.98) and preterm birth (5.1% vs 5.8%, aRR 0.94, 95% CI 0.90 to 0.99) (tables4  5).

**Table 4:** Association between COVID-19 vaccination during pregnancy and the risk of adverse neonatal outcomes

Outcomes	N (%)		Risk ratio (95% CI)	
	≥1 dose during pregnancy	No dose during pregnancy	Unadjusted	Adjusted*
Pregnancies with a live birth (cohort 2)	N=61 098	N=78 320		
Small for gestational age	5682 (9.3)	7996 (10.2)	0.91 (0.88 to 0.94)	0.94 (0.91 to 0.98)
NICU admission	2272/59 832† (3.8)	3353/76 437† (4.4)	0.86 (0.82 to 0.91)	0.91 (0.85 to 0.96)
Severe neonatal morbidity	1631/59 832† (2.7)	2455/76 437† (3.2)	0.85 (0.80 to 0.90)	0.91 (0.85 to 0.98)
Pregnancies with a live birth (cohort 2)	N=60 016	N=79 402		
Preterm birth <37 weeks	3079 (5.1)	4585 (5.8)	0.89 (0.85 to 0.93)	0.94 (0.90 to 0.99)
Spontaneous	2187 (3.6)	3244 (4.1)	0.89 (0.85 to 0.94)	0.95 (0.89 to 1.01)
Pregnancies with a live birth (cohort 2)	N=53 938	N=85 480		
Very preterm birth <32 weeks	319 (0.60)	613 (0.72)	0.83 (0.72 to 0.94)	1.04 (0.89 to 1.21)
Spontaneous	219 (0.41)	436 (0.51)	0.80 (0.68 to 0.94)	0.99 (0.82 to 1.19)

* Adjusted for maternal age at delivery, infant sex, maternal pre-existing conditions, healthcare provider status, SARS-CoV-2 positive test during pregnancy, social and material deprivation indexes, month and year of conception, Tdap vaccination during pregnancy and geographical areas using inverse probability of treatment weighting.

† For these issues, the information was only available in the newborn record linked with the mother record.

NICU, neonatal intensive care unit; Tdap, tetanus-diphtheria-acellular pertussis.

**Table 5:** Association between COVID-19 vaccination during pregnancy and the risk of stillbirth using a Cox regression model with time-varying exposure variable

Outcomes	Vaccinated		Unvaccinated		HR (95% CI)	
	Cases	Person-time at risk (days)	Cases	Person-time at risk (days)	Unadjusted	Adjusted*
Pregnancies with a live birth or stillbirth (cohort 1)						
Stillbirth	184	6 624 602	471	11 897 842	0.76 (0.64 to 0.91)	1.14 (0.94 to 1.39)

* Adjusted for maternal age at delivery, infant sex, maternal pre-existing conditions, healthcare provider status, SARS-CoV-2 positive test during pregnancy, social and material deprivation indexes, month and year of conception, Tdap vaccination during pregnancy and geographical areas using inverse probability of treatment weighting.

Tdap, tetanus-diphtheria-acellular pertussis.

### Subgroup and sensitivity analyses

In additional analyses, results remained similar regardless of the trimester of vaccination for the first dose, the vaccine product and the number of doses. The magnitude and the direction of the estimates were also similar after restricting the analysis to those who gave birth during the period of access to SARS-CoV-2 tests, excluding those with a SARS-CoV-2 infection during pregnancy, or varying the date of conception to address uncertainties. Although most subgroup analyses indicated that vaccination was not significantly associated with preterm births, we found a significant increased risk for individuals vaccinated with only one dose during pregnancy. Results did not change after excluding those unvaccinated throughout the entire study period from the unvaccinated group, except for the very preterm outcome, for which the estimate suggested a non-significant reduction in risk associated with vaccination (aRR 0.88, 95% CI 0.74 to 1.04 vs aRR in main analysis 1.04, 95% CI 0.89 to 1.21). Finally, we found a significant increased risk associated with COVID-19 vaccination for very preterm birth, using a Cox regression model and considering vaccination during pregnancy as a time-varying exposure (aHR 1.23, 95% CI 1.05 to 1.43 in the Cox model for very premature birth in online supplemental table S4 vs aRR 1.04, 95% CI 0.89 to 1.21 in table 4). (See all the subgroup and sensitivity analyses in online supplemental table S4 in the Supplement).

## Discussion

In this large study examining maternal and neonatal outcomes in more than 140 000 singleton pregnancies and including more than 60 000 individuals vaccinated with a mRNA vaccine during pregnancy, we found no evidence that COVID-19 vaccination during pregnancy was associated with a significant increased risk of chorioamnionitis, postpartum haemorrhage, caesarean delivery, maternal ICU admission, stillbirth, SGA, NICU admission, severe neonatal morbidity or premature births. Results did not differ according to the trimester of vaccination, the number of doses received during pregnancy and the vaccine product.

Our study adds to existing evidence that COVID-19 vaccination during pregnancy does not increase the risk of adverse maternal and neonatal outcomes. Our findings for maternal outcomes are consistent with conclusions reported in a systematic review and meta-analysis from Watanabe *et al* including 81 349 pregnant individuals who received at least one COVID-19 vaccination during pregnancy and 255 246 pregnant individuals not vaccinated.^39^ The pooled adjusted ORs were 0.95 (95% CI 0.83 to 1.07) for postpartum haemorrhage, 1.06 (95% CI 0.86 to 1.31) for chorioamnionitis and 1.05 (95% CI 0.93 to 1.21) for caesarean delivery. A Canadian study of 32 689 live births and stillbirths, including 18 491 individuals who received a third dose during pregnancy, reported aHRs for postpartum haemorrhage (1.01, 95% CI 0.89 to 1.16) and caesarean (0.90, 95% CI 0.87 to 0.94) that were similar to our estimates.^16^ Authors observed a significant reduction in the risk of chorioamnionitis (aHR 0.67, 95% CI 0.49 to 0.90) which was not observed in our study. Our findings of a reduced risk for NICU admission and SGA were also consistent with those reported by Wanatabe *et al*, with pooled adjusted ORs of 0.88 (95% CI 0.80 to 0.97) and 0.99 (95% CI: 0.04 to 1.04), respectively. Vaccination was also associated with a reduced risk of SGA (aHR of 0.93, 95% CI: 0.88 to 0.99) in a study from England and Wales, which included 865 654 pregnant individuals, of whom 60 875 received the first dose during pregnancy.^40^ Our results were also consistent with those reported in a study from Israel with 24 288 newborns, including 16 697 prenatally exposed to vaccination. They also reported no significant association with preterm birth (aRR 0.95, 95% CI 0.83 to 1.10) and SGA (aRR 0.97, 95% CI 0.87 to 1.08).^41^ For stillbirth, studies that accounted for immortal time bias reported results consistent with a reduced risk associated with vaccination. Fell *et al* found a statistically significant reduction of risk, while Magnus *et al* reported an aHR of 0.86 with 95% CI that included the null value.^18 33^ Although our estimate for stillbirth differs in direction, all estimates were consistent in showing that vaccination was not associated with an increased risk of stillbirth. A recent matched analysis of 276 confirmed stillbirths and 822 live births in the US Vaccine Safety Datalink found no association between COVID-19 vaccination during pregnancy and stillbirth (adjusted OR 1.02, 95% CI 0.76 to 1.37).^42^

Two studies that accounted for immortal time bias by treating vaccination as a time-varying exposure and using extended Cox regression models found that COVID-19 vaccination during pregnancy was not associated with an increased risk of preterm birth. The first study from Canada, which included 85 162 births and 43 099 individuals vaccinated during pregnancy, reported an aHR of 1.02 (95% CI 0.96 to 1.08) for preterm birth and 0.80 (95% CI 0.67 to 0.95) for very preterm birth.^33^ In the second study conducted in Sweden and Norway and involving 157 521 births—including 28 506 individuals vaccinated during pregnancy—the aHR for preterm birth, pooled across both countries, was 0.98 (95% CI 0.91 to 1.05).^18^ The positive association with vaccination (at least one dose) observed in our sensitivity analysis for very preterm birth should be interpreted with caution since it may reflect a type 1 error due to multiple testing or residual confounding by unmeasured characteristics and should be reassessed in future studies. There was also uncertainty in the estimate for very preterm birth due to the small number of events. Finally, we found a significant increased risk of preterm births among individuals vaccinated with only one dose during pregnancy, compared with those who were unvaccinated during pregnancy. However, individuals who gave birth prematurely may have been more likely to receive only one dose during pregnancy.

Strengths of this study include the large number of individuals vaccinated during pregnancy, as well as the variability in the timing of vaccination within the included cohort. This study also included periods during which vaccination was not limited to high-risk groups. The use of the provincial hospitalisation discharge database linked with the population-based vaccination registry ensures the cohort is representative of individuals who gave birth during the study period and provides accurate information on COVID-19 vaccine exposure during pregnancy. Nevertheless, this study has several limitations. Despite the availability of important potential confounders of the association between vaccination and outcomes and the adjustment done using propensity score weighting, we could not exclude the possibility of residual confounding. Indeed, we did not have information on some known risk factors for the outcomes of interest, such as parity, education level, ethnicity and smoking, which could be associated with vaccination. Although we did not have information on general attitudes regarding vaccination or health behaviours, Tdap vaccination during pregnancy was used as a proxy measure. Pregnant individuals with a more favourable health profile may be more likely to accept vaccination, a phenomenon called ‘healthy vaccine effect’, which could bias results downward.^43^ Due to the data source used, our cohort included only pregnancies that reached at least 20 weeks of gestation. This could have introduced a selection bias if COVID-19 vaccination in early pregnancy (less than 20 weeks of gestation) led to an increased risk of pregnancy loss before 20 weeks of gestation. Published studies found no evidence of a significant increased risk of miscarriage following vaccination, although the RR or OR was slightly higher than 1 in some studies.^44^,^46^ Non-differential misclassification of exposure and outcomes due to uncertainty for the date of conception was possible, but we observed similar results after varying the date of conception in sensitivity analyses. Non-differential outcomes misclassification is also possible due to errors in the codes assigned for some diagnostics.

Although the risk of hospitalisation and death among pregnant individuals has significantly decreased during the Omicron period, this group remains at higher risk of severe maternal complications, especially among those with severe COVID-19 symptoms and who are unvaccinated.^47 48^ Vaccination during pregnancy can effectively reduce this risk.^3^ Maternal vaccination could also protect young infants against severe disease during the first months of life through the transplacental passage of antibodies.^47^ We observed a reduction in the risk of caesarean delivery, maternal and neonatal ICU admission, SGA, severe morbidity index and preterm birth among individuals vaccinated during pregnancy compared with those who were not vaccinated. This is consistent with existing evidence suggesting that COVID-19 vaccination during pregnancy may reduce the risk of adverse maternal and neonatal outcomes.^48^ While severe COVID-19 is known to increase the risk of adverse pregnancy outcomes, our analysis suggests that infection prevention does not fully explain the reduced risk, since we adjusted for documented SARS-CoV-2 infection during pregnancy and results remained consistent in sensitivity analysis after excluding individuals with documented infection during pregnancy. Residual confounding is also possible, as we were not able to adequately account for confirmed SARS-CoV-2 infection during pregnancy due to changes in the screening strategy over the study period. In the sensitivity analysis using time-varying exposure and a Cox regression model, the protective effect of COVID-19 vaccines on preterm birth was annulled (aHR 1.01, 95% CI 0.96 to 1.06). Findings from this study, consistent with those of other studies, provide additional reassurance that COVID-19 vaccination during pregnancy is not associated with an increased risk of adverse maternal or neonatal outcomes. These findings would likely contribute to reducing vaccine hesitancy and increasing the acceptability of the COVID-19 vaccination during pregnancy. Future studies should include more pregnant individuals vaccinated during the first trimester to better assess outcomes with an earlier gestational onset or those plausibly associated with early pregnancy exposure. The comparison of different approaches for handling immortal time bias in estimating the effects of vaccination during pregnancy would be of interest.^49^ Finally, ongoing surveillance of the safety of maternal COVID-19 vaccination is essential, as additional doses of COVID-19 vaccine are recommended for pregnant individuals in many countries.

## Conclusions

Our findings, consistent with existing evidence, support that COVID-19 vaccination during pregnancy is not associated with an increased risk of adverse maternal and neonatal outcomes. Ongoing surveillance of the safety of maternal COVID-19 vaccination is essential to ensure the benefit-RR remains favourable in this population. Future studies should focus on outcomes plausibly associated with vaccination earlier in pregnancy, such as congenital anomalies.

## Supplementary material

10.1136/bmjopen-2025-106494 online supplemental file 1

## Data Availability

Data may be obtained from a third party and are not publicly available.
