# Supplementary material for: Safety of COVID-19 vaccines among pregnant individuals in Quebec, Canada: a population-based retrospective cohort study from the Canadian Immunization Research Network
Source: BMJ Open. 2025 Dec 23;15(12):e106494. doi: 10.1136/bmjopen-2025-106494 (PMC12730766; doi:10.1136/bmjopen-2025-106494)
Supplement: online supplemental file 1 [file bmjopen-15-12-s001.docx]

**Table S1 : Description of data sources used in the study**

| **Databases** | **Description** | **Utilization in the study** |
| --- | --- | --- |
| Maintenance et exploitation des données pour l’étude de la clientèle hospitalière (MED-ECHO) | This database contains data related to hospital stays that occurred in Quebec hospitals providing general and specialized care. These data, compiled by the hospitals, concern short-term care (both physical and psychiatric) and same-day surgeries.  We linked the mother’s hospital record to the newborn’s record (97%). | Was used to identify the study population using this algorithm: ICD-10-CA diagnostic codes starting with Z37 with a procedural code associated with a vaginal or caesarean delivery (starting with 5.MD.5 ou 5.MD.6) or with a diagnostic codes O10 to O16, O21 to O29, O30 to O46, O48, O60 to O75, O85 to O92, O95 or O98 à O99, with a 6^th^ term 1 or 2).  Also used to obtain information about gestational age at birth, maternal age at delivery, baby’s sex, birth weight and to define outcomes. For the latter we used information regarding diagnosis and interventions, as well as admission to neonatal intensive care unit. |
| Provincial Laboratory Database | This database is hosted by the Institut national de santé publique du Québec (INSPQ). It is integrated by NOSOTECH (which draws information from laboratory information systems and the provincial laboratory information system) and performs data standardization. It provides information on diagnostic tests for COVID-19 performed using RT-PCR. | Was used to identify confirmed cases of COVID-19 during pregnancy. |
| Quebec Vaccination Registry | The Quebec Vaccination Registry is a provincial computerized system in which all vaccines received by a person in Quebec must be recorded. This is a population-based registry, with sociodemographic data updated through Quebec universal public health insurance database, which covers approximately 97% of Quebecers.  More than 98% of the targeted cohort were successfully linked with the registry using unique Quebec health insurance number. | Was used to document maternal COVID-19 vaccination during pregnancy, including vaccine products, the number of doses and dates of vaccination. Maternal address, including postal code was extracted from this database. |
| Quebec Integrated Chronic Disease Surveillance System (QICDSS) | This database links patient-level records of these provincial health system administrative databases: the health insurance registry, the hospital inpatient and day surgery database, the vital statistics death database, the pharmaceutical services database.  (≥95% successfully matched with MED-ECHO) | Was used to identify pre-pregnancy medical conditions. |
| Material and social deprivation indices | These indices use Statistics Canada’s 2016 Census data to rank small areas from least deprived to most deprived according to education, employment, income and housing (proportion separated, divorced or widowed, living alone or in a single-parent family). The distribution of areas is then divided in quintiles, from 1 representing the population living in the least deprived areas, to 5 representing the one living in the most deprived areas. | Was used to obtain information regarding material and social deprivation indices. |

**Table S2: Variable definitions and ICD-10 diagnostic or CCI procedural codes used**

| **Variables** | **Definition** | **Data sources, ICD-10 or CCI codes** |
| --- | --- | --- |
| **Vaginal or cesarean delivery** |  | CIM-10 codes starting with Z37 with a procedural code associated with a vaginal or caesarean delivery (starting with 5.MD.5 or 5.MD.6) or with a diagnostic codes O10 to O16, O21 to O29, O30 to O46, O48, O60 to O75, O85 to O92, O95 or O98 à O99, with a 6^th^ term 1 or 2). |
| **Exposure** | | |
| COVID-19 vaccination | Receipt of at least one dose of mRNA COVID-19 vaccine during pregnancy. | Measured using doses of COVID-19 vaccine in the QVR and date of conception and date of delivery variables in MED-ECHO. |
| **Outcomes** | | |
| Chorioamnionitis | Infection and resulting inflammation of the chorion, amnion, amniotic fluid, placenta, decidua, fetus, or a combination | O4112, O4113, O4119 |
| Postpartum hemorrhage | Blood loss of ≥ 500 mL following  vaginal delivery or ≥ 1000 mL  following caesarean section. | O720, O721, O723 |
| Cesarean delivery | Surgical delivery by incision into the uterus. | 5.MD.60 |
| - Emergency cesarean delivery | Emergency caesarean delivery is performed to immediately intervene to improve maternal or fetal outcome for such indications as fetal distress, prolapsed cord, maternal hemorrhage from previa or trauma, uterine rupture, and complete placental abruption. | 5.MD.60 and one of the following:  VBAC code (O75.7)  Failed VBAC (O66.40, O66.49)  Medical induction of labor (5AC30ALI2, 5AC30CAI2, 5AC30GUI2, 5AC30HAI2, 5AC30YAI2, 5AC30YBI2, 5AC30ZZI2, 5AC30AP, or 5AC30)  Inducing or augmenting labor (5AC30AN, 5AC30AP, 5AC30CKA2, 5AC30CKBD, 5AC30CKI2, 5AC30CKW6, 5AC30HAI2, 5AC30YAI2, 5LD31)  Failed induction, abnormalities of forces of labor, long labor, obstructed labor and other codes indicating labor associated complications (O61-69)  Placental abruption (O45)  Uterine rupture (O71.1 – only if associated with spontaneous or induce labor)  Severe preeclampsia, HELLP syndrome or eclampsia (O14.1, O14.2, O15)  Failed forceps/vacuum (O66.5)  Chorioamnionitis (O4112, O4113, O4119)  Prelabour rupture of membrane (O42) |
| Stillbirth | Fetal death before the complete expulsion or extraction of products of conception after at least 20 weeks of pregnancy. | Z371, Z373, Z374, Z376, Z377, O364 |
| Small for gestational age (SGA) | Birth weight below the 10^th^ percentile of a reference population by sex and gestational age. | Measured using infant sex, gestational age, and birth weight variables in MED-ECHO. |
| Preterm birth | Birth before 37 weeks of gestation | Measured using gestational age variable in MED-ECHO. |
| - Spontaneous preterm labor | Spontaneous onset of labor or following prelabour premature rupture of membranes | O60101, O45, O4201, O4209, O4211, O4219, O422, O429 |
| - Medically indicated preterm delivery | Early delivery due to obstetric intervention. | O60301 or cesarean without spontaneous preterm labor (O60101, O60201). |
| Very preterm birth | Birth before 32 weeks of gestation | Measured using gestational age variable in MED-ECHO |
| - Spontaneous preterm labor | Spontaneous onset of labor or following prelabour premature rupture of membranes | O60101, O45, O4201, O4209, O4211, O4219, O422, O429 |
| - Medically indicated preterm delivery | Early delivery due to obstetric intervention. | O60301 or caesarean without spontaneous preterm labor (O60101, O60201). |
| Neonatal intensive care unit (NICU) | NICU admission during the hospitalisation for delivery (any indication) | Measured using variables in MED-ECHO. |
| Severe neonatal morbidity | Outcome used to identify infants with severe or medically significant morbidity., which includes 15 diagnostics and 7 procedures. |  |
| - Gestational age < 32 weeks | Birth before 32 weeks of gestation | Measured using gestational age variable in MED-ECHO |
| - Birth weight < 1500 | Birth weight less than 1500 grams | Measured using birth weight in MED-ECHO |
| - Respiratory distress | When baby's lungs are not fully developed and cannot provide enough oxygen, causing breathing difficulties. | P220 |
| - Seizures | Paroxysmal electroclinical phenomenon characterized by the transient occurrence of signs and symptoms due to an abnormal excessive or synchronous neuronal activity in the brain. | P90, R56 |
| - Interventricular hemorrhage (grades 2, 3 or 4) | Condition involving bleeding in the brain's fluid-filled areas. | P521, P522 |
| - Cerebral infarction | Neurologic deficits that result from focal cerebral ischemia associated with permanent brain infarction. | I63 |
| - Periventricular Leukomalacia | Characterized by the death of the brain's white matter due to softening of the brain tissue | P912 |
| - Birth trauma | Intracranial hemorrhage paralysis due to brachial plexus injury, skull or long bone fracture | P100 to P103, P130, P133, P140, P141 |
| - Hypoxic ischemic encephalopathy | Type of brain injury or damage that is caused by a lack of oxygen to the brain during neonatal period. | P915, P9181, P916 |
| - Necrotizing enterocolitis | Acute inflammatory disease of the intestine. | P77 |
| - Bronchopulmonary dysplasia | Chronic form of injury to the lungs caused by barotrauma and oxygen injury requiring mechanical ventilation. | P271 |
| - Sepsis/septicemia | Clinical syndrome defined by a systemic response to infection. | P36, A40, A415, A419, B951, B962 |
| - Pneumonia | Acute respiratory infection that affects the alveoli and distal airways. | P23, J12 to J18 |
| - Primary atelectasis | Pulmonary collapse, accompanied by hypoventilation; it can affect a lobe, segment, or all the lung, resulting in a decrease in the ventilation/perfusion ratio | P280 |
| - Respiratory failure | Occurs when the lungs fail to oxygenate the arterial blood adequately and/or fail to prevent carbon dioxide retention. | P285 |
| - Resuscitation | Set of interventions at the time of birth to support the establishment of breathing and circulation. | 1GZ30 |
| - Ventilatory support (mechanical ventilation and/or CPAP) | Methods of care that supports breathing. | 1GZ31 (without non-invasive approaches-1GZ31CB-ND, 1GZ31JA-NC, 1GZ31CB-EP) |
| - Central venous or arterial catheter | Arterial catheters are placed to facilitate frequent blood sampling and to closely monitor blood pressure.  Central venous catheters are used for many reasons including facilitating administration of certain medications, augmenting hemodynamic monitoring including determination of central venous oxygenation, and providing venous access when peripheral access is limited. | 1IS53, 1KV53 |
| - Transfusion of blood or blood products | Process of receiving blood or blood products intravenously. | 1LZ19HHU1A, 1LZ19HHU1J, 1LZ19HHU9A, 1LZ19HHU9J, 1LZ19HMU1, 1LZ19HMU9, 1LZ35HHT7, 1LZ35HAT7, 1LZ19HMU2, 1LZ19HHU2A, 1LZ19HHU2J |
| - Pneumothorax requiring an intercostal catheter | Surgical aseptic technique used to drain a pneumothorax. | 1GT33 |
| **Covariates** | | |
| Age at delivery | Age of the mother at the time of giving birth. | Measured using date of birth in MED-ECHO |
| Infant sex | Sex of the baby at delivery. | Measured using infant sex in MED-ECHO variable |
| Pre-pregnancy cardiac diseases | Identified from the QICDSS database with ICD-10 codes. | I50, G45, G46, I60-I69, I21, I22, I252, I441–I443, I456, I459, I47–I49, R000, R001, R008, T801, Z450, Z950, I05–I08, I091, I098, I34–I39, Q230–Q233, Q238, Q239, Z952, Z953, Z954, A520, I70x, I71, I72, I730, I731, I738, I739, I771, I790, K551, K558, K559, Z958, Z959, |
| Pre-pregnancy diabetes | Identified from the QICDSS database with ICD-10 codes. | E100, E101, E109, E110, E111, E119, E130, E131, E139, E140, E141, E149, E102, E1078, E112, E1178, E132, E1378, E142, E1478 |
| Pre-pregnancy hypertension | Identified from the QICDSS database with ICD-10 codes. | I10, I11–I13, I15, I674 |
| Pre-pregnancy hypothyroidism | Identified from the QICDSS database with ICD-10 codes. | E00, E01, E02, E03, E890 |
| Pre-pregnancy immunosuppression | Identified from the QICDSS database with ICD-10 codes. | B24, M05, M06, M08 |
| Pre-pregnancy obesity | Identified from the QICDSS database with ICD-10 codes. | E66 |
| Pre-pregnancy respiratory diseases | Identified from the QICDSS database with ICD-10 codes. | I278, I279, J40-J47, J60-J64, J65, J66, J67, J684, J701, J703, I26, I27, I280, I288, I289 |
| Health care worker | Includes all employees in the healthcare sector listed on the payroll of public and accredited private institutions or those members of the following professional orders: Quebec College of Physicians, Quebec Order of Nurses, Québec Order of Auxiliary Nurses, Quebec Order of Pharmacists, Quebec Order of Midwives, Quebec Professional Order of Respiratory Therapists. | Measured using the QVR matched with the lists of health care workers. |
| Pertussis vaccination | Receipt of pertussis vaccination during vaccination | Measured using the QVR and date of conception and date of delivery variables in MED-ECHO. |
| Geographical areas | Refers to the living environment, determined using postal code | Measured using maternal postal code in the QVR. |
| History of COVID-19 infection | Testing positive for SARS-CoV-2 during pregnancy | Measured using dates and results of COVID-19 tests in the PLD |
| Material deprivation index | Created using 3 indicators: 1) the proportion of the population aged 15 years and over without a high school diploma or equivalent; 2) the employment to population ratio for the population 15 years and over; 3) the average income of the population aged 15 years and over | Measured using Statistics Canada’s 2016 Census data available at the dissemination level area level and maternal postal code in the QVR. |
| Social deprivation index | Created using 3 indicators: 1) The proportion of the population aged 15 and over living alone; 2) The proportion of the population aged 15 and over who are separated, divorced or widowed; 3) The proportion of single-parent families. | Measured using Statistics Canada’s 2016 Census data available at the dissemination level area level and maternal postal code in the QVR. |
| Estimated date of conception | Refers to the estimated time of conception. | Measured using month and year of conception. Estimated by subtracting gestational age from the date of delivery (+ 2 weeks) available in MED-ECHO |

**Table S3: Predicted probability estimated from a logistic regression of receiving at least one dose of COVID-19 vaccine during pregnancy conditional on baseline characteristics^a^**

| **Characteristics** | Beta | Standard error |
| --- | --- | --- |
| **Intercept** | 1.22197 | 0.03086 |
| **Maternal age group, year** |  |  |
| 15-24 | -0.76891 | 0.02539 |
| 25-29 | -0.4034 | 0.01563 |
| 30-34 (ref) |  |  |
| 35-39 | 0.07347 | 0.01673 |
| 40-49 | -0.02911 | 0.02697 |
| **Infant sex, males vs females** | -0.01534 | 0.01245 |
| Unknown/missing | -0.00278 | 0.04038 |
| **Pre-pregnancy medical condition (1 vs 0)** |  |  |
| Cardiovascular diseases | 0.03914 | 0.04179 |
| Diabetes | 0.09546 | 0.0676 |
| Hypertension | -0.00425 | 0.05796 |
| Hypothyroidism | 0.06365 | 0.02153 |
| Immunosuppression | 0.19492 | 0.05811 |
| Obesity | 0.02661 | 0.02333 |
| Respiratory diseases | 0.1215 | 0.02387 |
| Unknown/missing^c^ | -0.98461 | 0.04894 |
| **Health care worker (1 vs 0)** | 0.28703 | 0.01767 |
| **Geographical areas** |  |  |
| Census metropolitan areas of Montreal (ref) |  |  |
| Others census metropolitan areas | -0.07447 | 0.01635 |
| Cities of 10 000 to 100 000 residents | -0.22959 | 0.02274 |
| Small cities and rural areas: less than 10 000 residents | -0.20009 | 0.01854 |
| Unknown/missing | -0.52751 | 0.11443 |
| **History of SARS-CoV-2 infection during pregnancy (1 vs 0)** | -0.35272 | 0.0204 |
| **Material deprivation index** |  |  |
| 1 (least deprived) (ref) |  |  |
| 2 | -0.27848 | 0.02039 |
| 3 | -0.43851 | 0.0206 |
| 4 | -0.60563 | 0.0211 |
| 5 (most deprived) | -0.79033 | 0.02157 |
| Unknown/missing | -0.5292 | 0.03139 |
| **Social deprivation index** |  |  |
| 1 (least deprived) (ref) |  |  |
| 2 | -0.10561 | 0.02038 |
| 3 | -0.13178 | 0.02028 |
| 4 | -0.14072 | 0.02058 |
| 5 (most deprived) | -0.16521 | 0.02101 |
| Unknown/missing | -0.5292 | 0.03139 |
| **Estimated date of conception** |  |  |
| 2020-07 to 2020-09 | -1.99928 | 0.03101 |
| 2020-10 to2020-12 | -1.01983 | 0.024 |
| 2021-01 to 2021-03 | -0.49343 | 0.02468 |
| 2021-04 to 2021-06 (ref) | -0.65596 | 0.02434 |
| 2021-07 to 2021-09 | -1.45287 | 0.02421 |
| 2021-10 to 2021-12 | -2.56542 | 0.0275 |
| 2022-01 to 2022-03 | -2.69289 | 0.02948 |
| 2022-04 to 2022-06 | -2.7793 | 0.0313 |

^a^ 1) we first estimated propensity score from a logistic regression, representing the predicted probability of receiving at least one dose of COVID-19 vaccine during pregnancy (Table S3). 2) we computed inverse probability of treatment weights derived from the propensity score (min=0.042501 max= 1 median= 0.514044 Q1=0.256154 Q3=1. 3) weights were stabilized and truncated to the 1^st^ and 99^th^ percentiles.4) inverse probability of treatment weighted risk ratio and 95% CI were computed using modified Poisson regression models with a robust, sandwich-type variance estimator.

**Figure S1: Standardized mean differences before and after weighting using inverse probability of exposure weights^a^**

**^
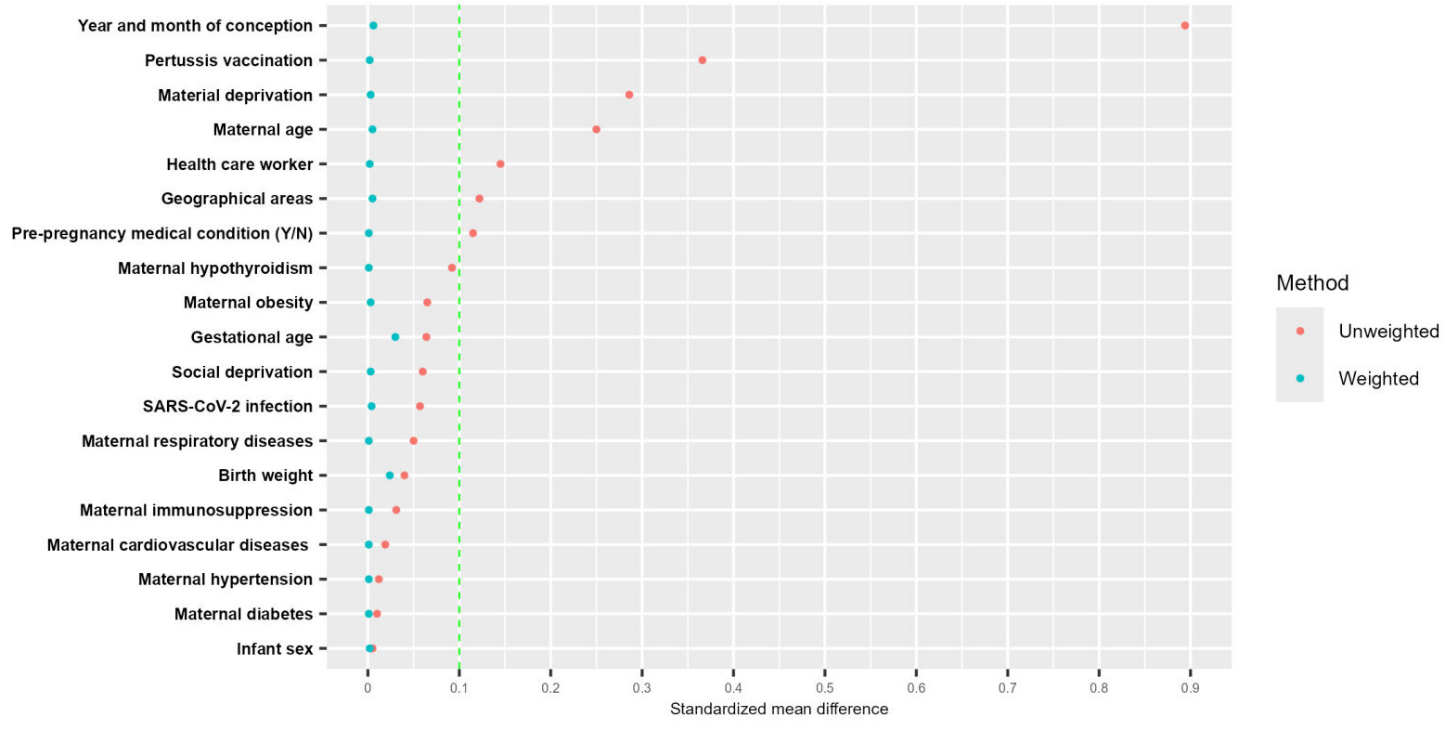
^**

^a^ Pre-pregnancy medical condition (Y/N), gestational age and birth weight not included in weighting.

**Figure S2: Distribution of SARS-CoV-2 infections during pregnancy^a^**

**
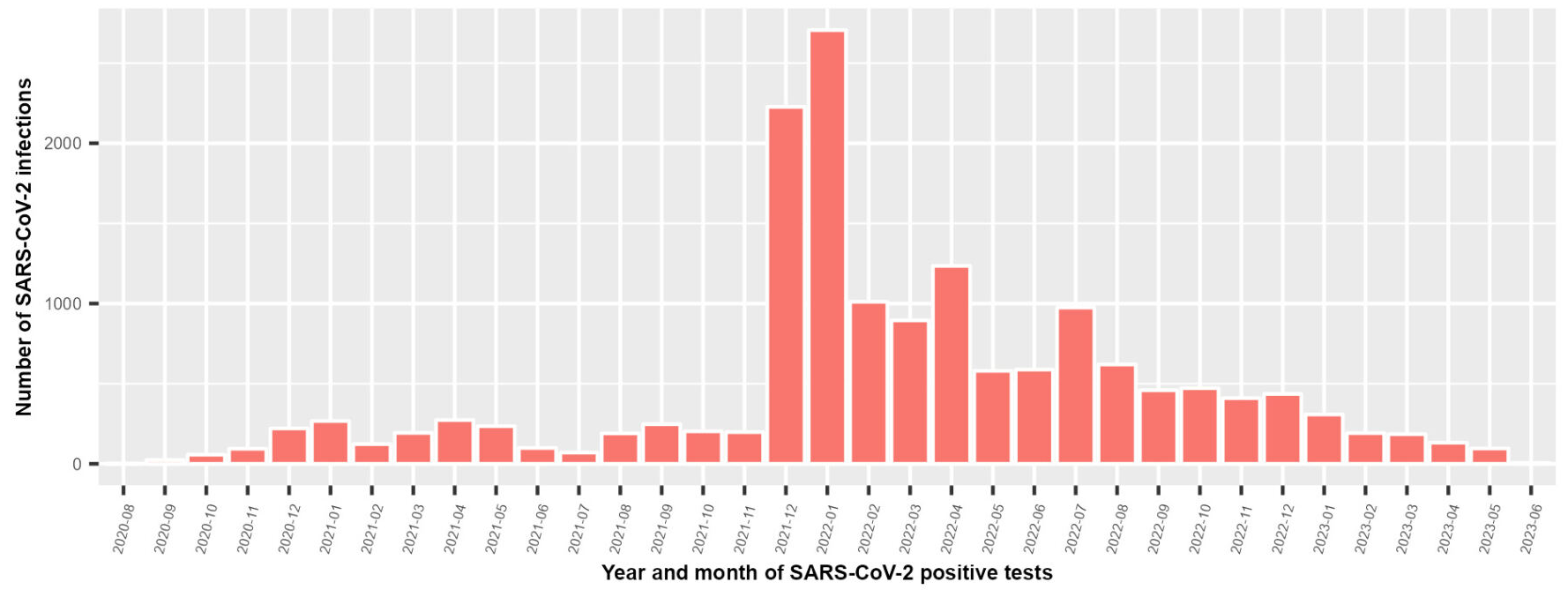
**

^a^ 1 pregnant individual with SARS-CoV-2 infection during pregnancy in August 2020 not represented in this figure

**Table S4: Association between COVID-19 vaccination during pregnancy and the risk of intrapartum complications and neonatal complications**

|  | **n/N (%)** | | **Risk ratio or hazard ratio (95% CI)** | |
| --- | --- | --- | --- | --- |
| **Outcomes** | **Vaccinated** | **Not vaccinated** | **Unadjusted** | **Adjusted^a^** |
| **Stillbirth using Poisson regression model** | | | | |
| Stillbirth | 184/61 282 (0.30%) | 471/78 791 (0.60%) | 0.50 (0.42-0.60) | 0.84 (0.69-1.02) |
| **Number of doses during pregnancy** | | | | |
| **Only one dose during pregnancy** | | | | |
| Chorioamnionitis | 1953/34 371 (5.7%) | 4472/78 791 (5.7%) | 1.00 (0.95-1.05) | 1.00 (0.94-1.06) |
| Postpartum hemorrhage | 3833/34 371 (11.1%) | 8757/78 791 (11.1%) | 1.00 (0.97-1.04) | 1.04 (1.00-1.08) |
| Cesarean delivery | 8929/34 371 (26.0%) | 21 222/78 791 (26.9%) | 0.96 (0.94-0.99) | 0.95 (0.924-0.97) |
| Maternal ICU admission | 108/34 371 (0.31%) | 315/78 791 (0.4%) | 0.79 (0.63-0.98) | 0.85 (0.67-1.08) |
| Stillbirth | 126/34 371 (0.37%) | 471/78 791 (0.60%) | 0.61 (0.50-0.75) | 0.97 (0.78-1.21) |
| Small for gestational age | 3199/34 245 (9.3%) | 7996/78 320 (10.2%) | 0.92 (0.88-0.95) | 0.94 (0.90-0.98) |
| Infant NICU admission | 1390/35 539 (4.1%) | 3353/76 437 (4.4%) | 0.95 (0.89-1.00) | 0.97 (0.91-1.04) |
| Severe neonatal morbidity index | 1009/33 539 (3.0%) | 2455/76 437 (3.2%) | 0.94 (0.87-1.01) | 1.01 (0.93-1.56) |
| Preterm birth | 1945/33 164 (5.9%) | 4585/79 402 (5.8%) | 1.02 (0.97-1.07) | 1.07 (1.01-1.14) |
| Very preterm birth | 236/27 459 (0.86%) | 613/85 480 (0.72%) | 1.20 (1.03-1.39) | 1.44 (1.22-1.71) |
| **Two doses during pregnancy** | | | | |
| Chorioamnionitis | 1362/25 563 (5.3%) | 4472/78 791 (5.7%) | 0.94 (0.89-1.00) | 0.99 (0.92-1.07) |
| Postpartum hemorrhage | 2660/25 563 (10.4%) | 8757/78 791 (11.1%) | 0.94 (0.90-0.98) | 0.99 (0.94-1.04) |
| Cesarean delivery | 6577/25 563 (25.7%) | 21 222/78 791 (26.9%) | 0.96 (0.93-0.98) | 0.95 (0.92-0.98) |
| Maternal ICU admission | 6577/25 563 (25.7%) | 21 222/78 791 (26.9%) | 0.81 (0.64-1.03) | 0.78 (0.57-1.05) |
| Stillbirth | 58/25 563 (0.23%) | 471/78 791 (0.60%) | 0.38 (0.29-0.50) | 0.69 (0.50-0.96) |
| Small for gestational age | 2373/25 505 (9.3%) | 7996/78 320 (10.2%) | 0.91 (0.87-0.95) | 0.96 (0.91-1.02) |
| Infant NICU admission | 837/24 976 (3.4%) | 3353/76 437 (4.4%) | 0.76 (0.71-0.82) | 0.80 (0.73-0.88) |
| Severe neonatal morbidity index | 605/24 976 (2.4%) | 2455/76 437 (3.2%) | 0.75 (0.69-0.82) | 0.79 (0.70-0.88) |
| Preterm birth | 1104/25 504 (4.3%) | 4585/79 402 (5.8%) | 0.75 (0.70-0.80) | 0.75 (0.69-0.81) |
| Very preterm birth | 83/25 131 (0.33%) | 613/85 480 (0.72%) | 0.46 (0.37-0.58) | 0.54 (0.41-0.71) |
| **≥ 3 doses during pregnancy** | | | | |
| Chorioamnionitis | 77/1348 (5.7%) | 4472/78 791 (5.7%) | 1.01 (0.81-1.25) | 0.93 (0.71-1.21) |
| Postpartum hemorrhage | 136/1348 (10.1%) | 8757/78 791 (11.1%) | 0.91 (0.77-1.07) | 0.94 (0.78-1.14) |
| Cesarean delivery | 332/1348 (24.6%) | 21 222/78 791 (26.9%) | 0.91 (0.83-1.01) | 0.88 (0.79-0.99) |
| Maternal ICU admission | 1/1348 (0.07%) | 315/78 791 (0.40%) | 0.19 (0.03-1.32) | 0.15(0.02,1.14) |
| Stillbirth | 0/1348 (0.0%) | 471/78 791 (0.60%) | NA | NA |
| Small for gestational age | 110/1348 (8.2%) | 7996/78 791 (10.2%) | 0.80 (0.67-0.96) | 0.85 (0.69-1.05) |
| Infant NICU admission | 45/1317 (0.0%) | 3353/76 437 (4.4%) | 0.78 (0.58-1.04) | 0.81 (0.60-1.08) |
| Severe neonatal morbidity index | 17/1317 (1.3%) | 2455/76 437 (3.2%) | 0.40 (0.25-0.65) | 0.41 (0.26-0.67) |
| Preterm birth | 30/1348 (2.2%) | 4585/79 401 (5.8%) | 0.39 (0.27-0.55) | 0.40 (0.28-0.59) |
| Very preterm birth | 0/1348 (0.0%) | 613/85 480 (0.72%) | NA | NA |
| **Trimester of vaccination** | | | | |
| **First dose during the first trimester** | | | | |
| Chorioamnionitis | 1016/19 442 (5.2%) | 4472/78 791 (5.7%) | 0.92 (0.86-0.98) | 0.97 (0.89-1.05) |
| Postpartum hemorrhage | 2119/19 442 (10.9%) | 8757/78 791 (11.1%) | 0.98 (0.94-1.03) | 1.02 (0.96-1.07) |
| Cesarean delivery | 5065/19 442 (26.1%) | 21222/78 791 (26.9%) | 0.97 (0.94-0.99) | 0.95 (0.92-0.98) |
| Maternal ICU admission | 60/19 442 (0.31%) | 315/78 791 (0.40%) | 0.77 (0.59-1.02) | 0.70 (0.51,0.97) |
| Stillbirth | 91/19 442 (0.47%) | 471/78 791 (0.60%) | 0.78 (0.63-0.98) | 1.03 (0.79-1.33) |
| Small for gestational age | 1801/19 351 (9.3%) | 7996/78 320 (10.2%) | 0.91 (0.87-0.96) | 0.92 (0.87-0.98) |
| Infant NICU admission | 737/18 958 (3.9%) | 3353/76 437 (4.4%) | 0.80 (0.61-1.06) | 0.72 (0.53-1.00) |
| Severe neonatal morbidity index | 553/19 351 (2.7%) | 2526/78 320 (3.2%) | 0.89 (0.82-0.96) | 0.88 (0.80-0.97) |
| Preterm birth | 1074/19 351 (5.6%) | 4585/79 402 (5.8%) | 0.96 (0.90-1.03) | 0.98 (0.90-1.05) |
| Very preterm birth | 126/19 351 (0.65%) | 613/85 840 (0.72%) | 0.91 (0.75-1.10) | 1.01 (0.81-1.27) |
| **First dose during the second trimester** | | | | |
| Chorioamnionitis | 1667/29 032 (5.7%) | 4472/78 791 (5.7%) | 1.01 (0.96-1.07) | 1.02 (0.96-1.09) |
| Postpartum hemorrhage | 3126/29 032 (10.8%) | 8757/78 791 (11.1%) | 0.97 (0.93-1.01) | 1.01 (0.96-1.06) |
| Cesarean delivery | 7483/29 032 (25.8%) | 21222/78 791 (26.9%) | 0.96 (0.94-0.98) | 0.94 (0.92-0.97) |
| Maternal ICU admission | 101/29 032 (0.35%) | 315/78 791 (0.40%) | 0.87 (0.70,1.09) | 0.88 (0.68,1.14) |
| Stillbirth | 85/29 032 (0.29%) | 471/78 791 (0.60%) | 0.49 (0.39-0.62) | 0.88 (0.68-1.14) |
| Small for gestational age | 2723/28 947 (9.4%) | 7996/78 320 (10.2%) | 0.92 (0.88-0.96) | 0.97 (0.91-1.00) |
| Infant NICU admission | 1123/28 344 (4.0%) | 3353/76 437 (4.4%) | 0.90 (0.85-0.97) | 0.94 (0.87-1.01) |
| Severe neonatal morbidity index | 826/28 344 (2.9%) | 2455/76 437 (3.2%) | 0.91 (0.840-0.98) | 0.96 (0.88-1.05) |
| Preterm birth | 1551/28 947 (5.4%) | 4585/79 401 (5.8%) | 0.93 (0.88-0.98) | 0.97 (0.91-1.03) |
| Very preterm birth | 179/28 947 (0.62%) | 613/85 480 (0.72%) | 0.86 (0.73-1.02) | 1.08 (0.89-1.30) |
| **First dose during the third trimester** | | | | |
| Chorioamnionitis | 709/12 808 (5.5%) | 4472/78 791 (5.7%) | 0.98 (0.90-1.05) | 0.98 (0.90-1.07) |
| Postpartum hemorrhage | 1384/12 808 (10.8%) | 8757/78 791 (11.1%) | 0.97 (0.92-1.03) | 1.03 (0.97-1.10) |
| Cesarean delivery | 3290/12 808 (25.7%) | 21222/78 791 (26.9%) | 0.95 (0.92-0.98) | 0.94 (0.91-0.97) |
| Maternal ICU admission | 101/12 808 (0.35%) | 315/78 791 (0.40%) | 0.87 (0.70-1.09) | 0.88 (0.68-1.14) |
| Stillbirth | 8/12 808 (0.06%) | 471/78 791 (0.60%) | 0.10 (0.05-0.21) | 0.26 (0.13-0.53) |
| Small for gestational age | 1158/12 800 (9.1%) | 7996/78 320 (10.2%) | 0.89 (0.84-0.94) | 0.94 (0.88-1.00) |
| Infant NICU admission | 412/12 530 (3.3%) | 3353/76 437 (4.4%) | 0.75 (0.68-0.83) | 0.86 (0.77-0.96) |
| Severe neonatal morbidity index | 262/12 530 (2.1%) | 2455/76 437 (3.2%) | 0.65 (0.57-0.74) | 0.71 (0.62-0.81) |
| Preterm birth | 454/11 718 (3.9%) | 4585/79 402 (5.7%) | 0.67 (0.61-0.74) | 0.77 (0.69-0.85) |
| Very preterm birth | 14/5640 (0.25%) | 613/85 480 (0.72%) | 0.35 (0.20-0.59) | 0.46 (0.27-0.80) |
| **Type of vaccine received** | | | | |
| **BNT162b2 only** | | | | |
| Chorioamnionitis | 2397/43 137 (5.6%) | 4472/78 791 (5.7%) | 0.98 (0.93-1.03) | 0.98 (0.93-1.04) |
| Postpartum hemorrhage | 4643/43 137 (10.8%) | 8757/78 791 (11.1%) | 0.97 (0.94-1.00) | 1.02 (0.98-1.06) |
| Cesarean delivery | 10976/43 137 (25.4%) | 21222/78 791 (26.9%) | 0.95 (0.93-0.96) | 0.94 (0.92-0.96) |
| Maternal ICU admission | 116/43 137 (0.27%) | 315/78 791 (0.40%) | 0.68 (0.55-0.84) | 0.71 (0.56-0.91) |
| Stillbirth | 131/43 137 (0.30%) | 471/78 791 (0.60%) | 0.5 (0.42-0.62) | 0.93 (0.74-1.15) |
| Small for gestational age | 3911/43 006 (9.1%) | 7996/78 320 (10.2%) | 0.89 (0.86-0.92) | 0.91 (0.87-0.95) |
| Infant NICU admission | 4913/42 127 (3.7%) | 3353/76 437 (4.4%) | 0.84 (0.80-0.90) | 0.91 (0.85-0.97) |
| Severe neonatal morbidity index | 1168/42 127 (2.8%) | 2455/76 437 (3.2%) | 0.86 (0.81-0.93) | 0.92 (0.85-0.99) |
| Preterm birth | 2170/42 192 (5.1%) | 4585/79 402 (5.8%) | 0.89 (0.85-0.94) | 0.95 (0.89-1.00) |
| Very preterm birth | 231/37 573 (0.61%) | 613/85 480 (0.72%) | 0.86 (0.74-1.00) | 1.05 (0.89-1.25) |
| **mRNA-1273 only** | | | | |
| Chorioamnionitis | 876/16 086 (5.5%) | 4472/78 791 (5.7%) | 0.96 (0.89-1.03) | 1.01 (0.94-1.09) |
| Postpartum hemorrhage | 1773/16 086 (11.0%) | 8757/78 791 (11.1%) | 0.99 (0.95-1.04) | 1.04 (0.98-1.09) |
| Cesarean delivery | 4286/16 086 (26.6%) | 21222/78 791 (26.9%) | 1.00 (0.96-1.02) | 0.95 (0.92-0.98) |
| Maternal ICU admission | 69/16 086 (0.43%) | 315/78 791 (0.40%) | 1.07 (0.83-1.39) | 0.99 (0.74-1.32) |
| Stillbirth | 51/16 086 (0.32%) | 471/78 791 (0.60%) | 0.53 (0.40-0.71) | 0.76 (0.56-1.03) |
| Small for gestational age | 1579/16 035 (9.9%) | 7996/78 320 (10.2%) | 0.97 (0.92-1.02) | 1.01 (0.95-1.06) |
| Infant NICU admission | 644/15 688 (4.1%) | 3353/76 437 (4.4%) | 0.94 (0.86-1.02) | 0.92 (0.84-1.00) |
| Severe neonatal morbidity index | 419/15 688 (2.7%) | 2455/76 437 (3.2%) | 0.83 (0.75-0.92) | 0.90 (0.81-1.01) |
| Preterm birth | 851/15 767 (5.4%) | 4585/79 402 (5.8%) | 0.94 (0.87-1.00) | 0.96 (0.89-1.04) |
| Very preterm birth | 86/14 313 (0.60%) | 613/85480 (0.72%) | 0.84 (0.67-1.05) | 0.97 (0.76-1.24) |
| **Mixed mRNA** | | | | |
| Chorioamnionitis | 119/2059 (5.8%) | 4472/78 791 (5.7%) | 1.02 (0.85-1.22) | 1.06 (0.84-1.27) |
| Postpartum hemorrhage | 213/2059 (10.3%) | 8757/78 791 (11.1%) | 0.93 (0.82-1.06) | 0.98 (0.86-1.11) |
| Cesarean delivery | 576/2059 (28.0%) | 21,222/78 791 (26.9%) | 1.04 (0.97-1.11) | 1.00 (0.936-1.08) |
| Maternal ICU admission | 6/2059 (0.29%) | 315/78 791 (0.40%) | 0.73 (0.33-1.63) | 0.69 (0.30-1.56) |
| Stillbirth | 2/2059 (0.10%) | 471 /78 791 (0.60%) | 0.16 (0.04-0.65) | 0.24 (0.06-0.95) |
| Small for gestational age | 192/2057 (9.3%) | 7996/78 320 (10.2%) | 0.91 (0.80-1.05) | 0.92 (0.80-1.06) |
| Infant NICU admission | 68/2017 (3.4%) | 3353/76 437 (4.4%) | 0.77 (0.61-0.97) | 0.77 (0.60-0.97) |
| Severe neonatal morbidity index | 44/2017 (2.2%) | 2455/76 437 (3.2%) | 0.68 (0.51-0.91) | 0.72 (0.53-0.97) |
| Preterm birth | 58/2057 (2.8%) | 4585/79 402 (5.8%) | 0.49 (0.38-0.630) | 0.50 (0.38-0.65) |
| Very preterm birth | 2/2052 (0.10%) | 613/85 480 (0.72%) | 0.14 (0.03-0.54) | 0.15 (0.04-0.63) |
| **Timing of pregnancy^b^** | | | | |
| **Births before January 2022** | | | | |
| Chorioamnionitis | 1398/25 774 (5.4%) | 1190/22 077 (5.4%) | 1.01 (0.93-1.09) | 0.98 (0.90-1.06) |
| Postpartum hemorrhage | 2655/25 774 (10.3%) | 2293/22 077 (10.4%) | 0.99 (0.94-1.05) | 1.00 (0.95-1.06) |
| Cesarean delivery | 6652/25 774 (25.8%) | 5798/22 077 (26.3%) | 0.98 (0.95-1.01) | 0.95 (0.92-0.98) |
| Maternal ICU admission | 81/25 774 (0.31%) | 84/22 077 (0.38%) | 0.83 (0.61,1.12) | 0.82 (0.59-1.14) |
| Stillbirth | 90/25 774 (0.35%) | 106/22 077 (0.48%) | 0.73 (0.55-0.96) | 0.88 (0.65-1.19) |
| Small for gestational age | 2284/25 684 (8.9%) | 2134/21 971 (9.7%) | 0.92 (0.87-0.97) | 0.92 (0.87-0.98) |
| Infant NICU admission | 940/25 133 (3.7%) | 803/21 452 (3.7%) | 1.00 (0.91-1.10) | 0.95 (0.86-1.05) |
| Severe neonatal morbidity index | 770/25 133 (3.1%) | 700/21 452 (3.3%) | 0.94 (0.85-1.04) | 0.96 (0.86-1.07) |
| Preterm birth | 1393/24 830 (5.6%) | 1217/22 825 (5.3%) | 1.05 (0.98-1.13) | 0.95 (0.88-1.03) |
| Very preterm birth | 180/20 568 (0.88%) | 162/27 087 (0.60%) | 1.46 (1.18-1.81) | 1.20 (0.95-1.51) |
| **Conception date ≥ January 2022.** | | | | |
| Chorioamnionitis | 477/7782 (6.1%) | 2077/34 973 (5.9%) | 1.03 (0.94-1.14) | 1.02 (0.93-1.13) |
| Postpartum hemorrhage | 896/7782 (11.5%) | 4130/34 973 (11.8%) | 0.98 (0.91-1.04) | 1.03 (0.96-1.11) |
| Cesarean delivery | 2070/7782 (26.6%) | 9724/34 973 (27.8%) | 0.96 (0.92-1.00) | 0.90 (0.87-0.94) |
| Maternal ICU admission | 21/7782 (0.27%) | 151/34 973 (0.43%) | 0.63 (0.40-0.99) | 0.69 (0.43-1.10) |
| Stillbirth | 26/7782 (0.33%) | 266/34 973 (0.76%) | 0.44 (0.29-0.66) | 0.75 (0.50-1.14) |
| Small for gestational age | 807/7756 (10.4%) | 3628/34 707 (10.5%) | 1.00 (0.93-1.07) | 1.01 (0.94-1.09) |
| Infant NICU admission | 295/7591 (3.9%) | 1649/33 866 (4.9%) | 0.80 (0.71-0.90) | 0.81 (0.71-0.91) |
| Severe neonatal morbidity index | 171/7591 (2.3%) | 1126/33 866 (3.3%) | 0.68 (0.58-0.79) | 0.74 (0.62-0.87) |
| Preterm birth | 373/7617 (4.9%) | 2178/34 846 (6.3%) | 0.78 (0.70-0.87) | 0.87 (0.78-0.97) |
| Very preterm birth | 35/6666 (0.53%) | 319/35 797 (0.89%) | 0.59 (0.42-0.83) | 0.83 (0.58-1.19) |
| **Excluding individuals with a SARS-CoV-2 infection during pregnancy** | | | | |
| Chorioamnionitis | 3102/55 357 (5.6%) | 3967/69 797 (5.7%) | 0.99 (0.94-1.03) | 1.01 (0.96-1.06) |
| Postpartum hemorrhage | 6024/55 357 (10.9%) | 7723/69 797 (11.1%) | 0.98 (0.95-1.02) | 1.04 (1.00-1.08) |
| Cesarean delivery | 14256/55 357 (25.8%) | 18702/69 797 (26.8%) | 0.96 (0.94-0.98) | 0.94 (0.92-0.96) |
| Maternal ICU admission | 169/55 357 (0.31%) | 258/69 797 (0.37%) | 0.83 (0.68-1.00) | 0.86 (0.68-1.08) |
| Stillbirth | 169/55 357 (0.31%) | 427/69 797 (0.61%) | 0.50 (0.42-0.60) | 0.84 (0.68-1.03) |
| Small for gestational age | 5146/55 188 (9.3%) | 7129/69 370 (10.3%) | 0.91 (0.88-0.94) | 0.94 (0.91-0.98) |
| Infant NICU admission | 2046/54 025 (3.8%) | 2989/67 645 (4.4%) | 0.86 (0.81-0.91) | 0.90 (0.84-0.96) |
| Severe neonatal morbidity index | 1477/54 025 (2.7%) | 2152/67 645 (3.2%) | 0.86 (0.81-0.92) | 0.92 (0.86-1.00) |
| Preterm birth | 2758/54 190 (5.1%) | 4021/70 368 (5.7%) | 0.89 (0.85-0.93) | 0.94 (0.89-0.99) |
| Very preterm birth | 298/48 565 (0.61%) | 549/75 993 (0.72%) | 0.85 (0.74-0.98) | 1.05 (0.89-1.23) |
| **Unexposed group excluding unvaccinated individuals during the study period** | | | | |
| Chorioamnionitis | 3392/61 282 (5.5%) | 3350/58234 (5.8%) | 0.96 (0.99-1.01) | 0.98 (0.93-1.03) |
| Postpartum hemorrhage | 6629/61 282 (10.8%) | 6614/58 234 (11.4%) | 0.95 (0.92-0.98) | 1.03 (0.99-1.07) |
| Cesarean delivery | 15838/61 282 (25.8%) | 15866/58 234 (27.3%) | 0.95 (0.93-0.97) | 0.92 (0.90-0.94) |
| Maternal ICU admission | 192/61 282 (0.31%) | 212/58 234 (0.36%) | 0.86 (0.71-1.05) | 0.83 (0.66-1.05) |
| Stillbirth | 184/61 282 (0.30%) | 351/58 234 (0.60%) | 0.50 (0.42-0.60) | 0.71 (0.57-0.88) |
| Small for gestational age | 5682/61 098 (9.3%) | 5654/57 883 (0.36%) | 0.95 (0.92-0.99) | 0.99 (0.95-1.03) |
| Infant NICU admission | 2272/59 832 (3.8%) | 2476/56 651 (4.4%) | 0.87 (0.82-0.92) | 0.88 (0.82-0.94) |
| Severe neonatal morbidity index | 1631/59 832 (2.7%) | 1839/56 651 (3.3%) | 0.84 (0.79-0.90) | 0.86 (0.80-0.93) |
| Preterm birth | 3079/60 016 (5.1%) | 3438/58 965 (5.8%) | 0.88 (0.84-0.92) | 0.91 (0.86-0.96) |
| Very preterm birth | 319/53 938 (0.60%) | 476/65 043 (0.73%) | 0.81 (0.70-0.93) | 0.88 (0.74-1.04) |
| **Vaccine exposed if vaccinated ≤ 2 weeks before the date of conception** | | | | |
| Chorioamnionitis | 3510/63 588 (5.5%) | 4354/76 485 (5.7%) | 0.97 (0.93-1.01) | 0.99 (0.94-1.04) |
| Postpartum hemorrhage | 6878/63 588 (10.8%) | 8508/76 485 (11.1%) | 0.97 (0.94-1.00) | 1.02 (0.99-1.06) |
| Cesarean delivery | 16464/63 588 (25.9%) | 20596/76 485 (26.9%) | 0.96 (0.95-0.98) | 0.94 (0.92-0.96) |
| Maternal ICU admission | 200/63 588 (0.31%) | 307/76 485 (0.40%) | 0.78 (0.66-0.94) | 0.78 (0.64-0.96) |
| Stillbirth | 193/63 588 (0.30%) | 462/76 485 (0.60%) | 0.50 (0.43-0.59) | 0.83 (0.69-1.01) |
| Small for gestational age | 5884/63 395 (9.3%) | 7794/76 023 (10.3%) | 0.91 (0.88-0.94) | 0.93 (0.90-0.97) |
| Infant NICU admission | 2368/62 088 (3.8%) | 3257/74 181 (4.4%) | 0.87 (0.83-0.92) | 0.91 (0.85-0.96) |
| Severe neonatal morbidity index | 1696/62 088 (2.7%) | 2390/74 181 (3.2%) | 0.85 (0.80-0.90) | 0.91 (0.85-0.98) |
| Preterm birth | 3192/60 346 (5.3%) | 4472/79 072 (5.7%) | 0.94 (0.90-0.98) | 0.98 (0.93-1.03) |
| Very preterm birth | 336/53 695 (0.63%) | 596/85 723 (0.70%) | 0.90 (0.79-1.03) | 1.11 (0.95-1.30) |
| **Vaccine exposed if vaccinated ≥ 2 weeks after the date of conception** | | | | |
| Chorioamnionitis | 3275/59 329 (5.5%) | 4589/80744 (5.7%) | 0.97 (0.93-1.02) | 0.99 (0.94-1.04) |
| Postpartum hemorrhage | 6409/59 329 (10.8%) | 8977/80744 (11.1%) | 0.97 (0.94-1.00) | 1.02 (0.99-1.06) |
| Cesarean delivery | 15329/59 329 (25.8%) | 21731/80744 (26.9%) | 0.96 (0.94-0.98) | 0.94 (0.92-0.96) |
| Maternal ICU admission | 184/59 329 (0.31%) | 323/80744 (0.40%) | 0.76 (0.65,0.93) | 0.79 (0.64,0.98) |
| Stillbirth | 175/59 329 (0.29%) | 480/80744 (0.59%) | 0.50 (0.42-0.59) | 0.84 (0.69-1.02) |
| Small for gestational age | 5503/59 154 (9.3%) | 8175/80264 (10.2%) | 0.91 (0.88-0.94) | 0.94 (0.91-0.98) |
| Infant NICU admission | 2203/57 924 (3.8%) | 3422/78 345 (4.4%) | 0.87 (0.83-0.92) | 0.92 (0.86-0.98) |
| Severe neonatal morbidity index | 1574/57 924 (2.7%) | 2512/78 345 (3.2%) | 0.85 (0.80-0.90) | 0.91 (0.85-0.98) |
| Preterm birth | 2970/59 047 (5.0%) | 4694/80 371 (5.8%) | 0.86 (0.82-0.90) | 0.92 (0.88-0.97) |
| Very preterm birth | 305/54 539 (0.56%) | 627/84 879 (0.74%) | 0.76 (0.66-0.87) | 0.98 (0.83-1.14) |
| **Term births only** | | | | |
| Small for gestational age | 5393/58 019 (9.3%) | 7566/73 735 (10.3%) | 0.91 (0.88-0.94) | 0.93 (0.90-0.97) |
| Infant NICU admission | 125/56 818 (2.7%) | 187/71 988 (2.9%) | 0.92 (0.86-0.98) | 0.94 (0.87-1.01) |
| **Mother considered unexposed if vaccinated exclusively within the last 2 weeks of pregnancy** | | | | |
| Small for gestational age | 5578/59 895 (9-3%) | 8100/79 523 (10.2%) | 0.91 (0.89-0.94) | 0.94 (0.91-0.98) |
| **Cox model for prematurity** | | | | |
| Premature birth <37 w | 3079/5 719 220 (5.4 per 10 000 pregnancy days at risk) | 4585/10 730 268 (4.3 per 10 000 pregnancy days at risk) | 0.98 (0.93-1.02) | 1.01 (0.96-1.06) |
| Very premature birth <32 w | 319/3 763 820 (0.85 per 10 000 pregnancy days at risk) | 613/7 919 783 (0.77 per 10 000 pregnancy days at risk) | 1.00 (0.88-1.15) | 1.23 (1.05-1.43) |

^a^ Adjusted for maternal age at delivery, infant sex, maternal pre-existing conditions, healthcare provider status, SARS-CoV-2 positive test during pregnancy, social and material deprivation indexes, month and year of conception, Tdap vaccination during pregnancy and geographical areas using inverse probability of treatment weighting. The propensity scores were estimated for each sensitivity analysis.

^b^ Individuals with a date of conception before January 2022 and a birth date ≥ January 2022 were excluded from this analysis.
