# Supplementary material for: Safety of COVID-19 vaccines among pregnant individuals in Quebec, Canada: a population-based retrospective cohort study from the Canadian Immunization Research Network
Source: BMJ Open. 2025 Dec 23;15(12):e106494. doi: 10.1136/bmjopen-2025-106494 (PMC12730766; doi:10.1136/bmjopen-2025-106494)
Supplement: Supplementary file 2 [file bmjopen-2025-106494.reviewer_comments.pdf]

PEER REVIEW HISTORY

BMJ Open publishes all reviews undertaken for accepted manuscripts. Reviewers are asked to complete a checklist review form and are provided with free text boxes to elaborate on their assessment. These free text comments are reproduced below.

ARTICLE DETAILS

Title (Provisional)

Safety of COVID-19 Vaccines Among Pregnant Individuals in Quebec, Canada: a Population-based Retrospective Cohort Study from the Canadian Immunization Research Network

Authors

Kiely, Marilou; Diendere, Ella; Dialahy, Isaora; Perrault-Sullivan, Gentiane; Brousseau, Nicholas; Wei, Shu Qin; Talbot, Denis; Boutin, Amélie; Quach, Caroline; Jorgensen , Sarah; Boucoiran, Isabelle

---

VERSION 1 - REVIEW

**Reviewer**	**1**
**Name**	**Kelson, Zoe**
**Affiliation**	**University of Exeter, Mathematics**
**Date**	**22-Jul-2025**
**COI**	**None**

This population-based retrospective cohort study aims to estimate the association between maternal COVID-19 vaccination during pregnancy and adverse neonatal and maternal outcomes.

Reviewer comments:

"We followed the RECORD (REporting of studies Conducted using Observational Routinely-collected health Data) statement and guidelines for observational studies of COVID-19 vaccination during pregnancy"

Thanks for providing a copy of the RECORD checklist.

"Province of Quebec, Canada, from May 1, 2021, to June 30, 2023. " [Abstract]

and

"conducted a retrospective population-based cohort study among individuals who gave birth between May 1, 2021, and June 30, 2023, in hospitals in Quebec."

and

"About 97% of births in Quebec occur in a hospital. Hospital delivery records of mothers were linked with the corresponding 12 newborn records (97% successfully linked). We deterministically linked the cohort with 03 four other databases, using the mothers' unique Quebec health insurance number:... "

and

"Among 140 073 singleton pregnancies resulting in live birth or stillbirth, 61 282 individuals (43.8%) received at least one dose of mRNA COVID-19 vaccine during pregnancy." [Abstract]

and

"There were 159 394 pregnant individuals who gave birth in hospital in Québec from May 22 1, 2021, to June 30, 2023 (Figure 1). After exclusions, there were 140 073 singleton pregnancies that resulted in live births or stillbirths (cohort 1), and 61 282 individuals (43.8%) received at least one dose of a mRNA COVID-19 vaccine during pregnancy"

Can the authors please confirm whether all hospitals in Quebec were included in this study?

Can the authors please comment on whether the final analysed cohort can be considered to be representative?

"The study used health administrative data, and no additional information was collected from participants"

The Tables show missing data (with some variables up to 7.4% missing).

Can the authors please clarify how this missing data was handled in the analysis?

Can the authors please comment on whether the missing data can be considered to be missing at random?

"The following variables were considered potential confounders for the association between vaccination and maternal and neonatal outcomes: maternal age at delivery (categorized), infant sex, pre-pregnancy medical conditions (cardiovascular diseases, diabetes, hypertension, hypothyroidism, immunosuppression, obesity and respiratory diseases), health care worker status, tetanus-diphtheria-acellular pertussis (Tdap) vaccination during pregnancy (proxy for health behaviors), history of at least one positive SARS-CoV-2 test during pregnancy, geographical areas, month and year of conception and area-level social and material deprivation indices (See Table S2 in the Supplement for a description of each covariate)."

Can the authors please consider assessing and adjusting for lockdowns and responses to the pandemic at different stages as a potential confounder? For instance, both vaccination and caesarean rates may be influenced by pandemic timeline/restrictions in place?

"We used robust Poisson regression models to estimate adjusted risk ratios (aRRs) for chorioamnionitis, postpartum hemorrhage, cesarean delivery, preterm birth, very preterm birth, small for gestational age (SGA), maternal and 17 neonatal admission to intensive care

unit (ICU, NICU) and severe neonatal morbidity. We used a Cox regression model with a time varying exposure variable to estimate adjusted hazard ratios (aHRs) for stillbirth. Propensity score weighting was used to adjust for potential confounding." [Abstract]

and

"To control for potential 72 confounding for the association between vaccination and outcomes, we computed inverse probability of treatment weights derived from the propensity score. This score represented the predicted probability, estimated from a logistic regression, of receiving at least one dose of mRNA COVID-19 vaccine during pregnancy conditional on observed baseline characteristics. Weights were stabilized and truncated to the 1st and 99th percentiles to address the problem of large weight that can increase variability in the effect being 178 estimated. Unadjusted and inverse probability of treatment weighted risk ratios (RR) and 95% confidence (CI) were computed using modified Poisson regression models with a robust, sandwich-type variance estimator to account for weight-induced dependencies...

Adjusted hazard ratios (HR) with 95% CI was then computed using a weighted Cox-proportional hazard regression model with gestational age in days as the time scale and follow-up time beginning at 20 weeks until the end of pregnancy or the occurrence of stillbirth. Vaccination during pregnancy was considered a time-varying exposure, with pregnant individuals contributing to both exposed and unexposed time during pregnancy. The validation of proportional hazards assumption was based on the Schoenfeld residuals test"

Appropriate and rigorous modelling methods have been applied by the authors.

Can the propensity model please be shown in full in the supplementary material?

"Statistical significance was set at 5% and the study was powered at over 80% to detect a 1% absolute increase following vaccination for rare outcomes such as stillbirth"

The authors suitably communicate their assessment of sample size and associated study power.

"We performed the following subgroup and sensitivity analyses to test the robustness of our results..."

A number of insigthful additional analyses help to explore the study findings.

"Table 1: Unweighted baseline characteristics of the total study population and by maternal COVID-19 vaccination during pregnancy "

Whilst the authors note in the study limitation that 'we did not have information on some known risk factors for the outcomes of interest, such as parity, education level, ethnicity and smoking, which could be associated with vaccination', is information on maternal BMI available?

"After propensity score weighting, the distribution of baseline characteristics was similar between vaccinated and unvaccinated individuals, with all standardized difference less than 0.1 (Figure S1 in the Supplement)."

Can unweighted and weighted characteristics, and the change in standardised differences between vaccination groups, please be shown in the same table (i.e. Table 1)?

"Although we adjusted for potential confounders, residual confounding cannot be excluded. " [strengths and limitations of the study]

and

"Nevertheless, this study has several limitations. Despite the availability of important potential confounders of the association between vaccination and outcomes and the adjustment done using propensity score weighting, we could not exclude the possibility of residual confounding. Indeed, we did not have information on some known risk factors for the outcomes of interest, such as parity, education level, ethnicity and smoking, which could be associated with vaccination. Although we did not have information on general attitudes regarding vaccination or health behaviors, Tdap vaccination during pregnancy was used as a proxy measure. Pregnant individuals with a more favorable health profile may be more likely to accept vaccination, a phenomenon called "healthy vaccine effect", which could bias results downward. Due to the data source used, our cohort included only pregnancies that reached at least 20 weeks of gestation. This could have introduced a selection bias if COVID-19 vaccination in early pregnancy (less than 20 weeks of gestation) led to an increased risk of pregnancy loss before 20 weeks of gestation. Published studies found no evidence of a significant increased risk of miscarriage following vaccination, although the risk ratio or odds ratio were slightly higher than 1 in some studies44–46. Non-differential misclassification of exposure and outcomes due to uncertainty for the date of conception was possible, but we observed similar results after varying the date of conception in sensitivity analyses. Non-differential outcomes misclassification is also possible due to errors in the codes assigned for some diagnostics. "

The authors provide a discussion on the study limitations.

Thanks for providing a copy of the Research Protocol, which aligns with the methodology reported in the main article.

**Reviewer**	**2**
**Name**	**Cambou, Mary C.**
**Affiliation**	**University of California Los Angeles David Geffen School of Medicine**
**Date**	**01-Aug-2025**
**COI**	**None**

This is an outstanding paper that contributes to the literature on the safety of COVID-19 mRNA vaccines in pregnancy. In order to estimate the association between receipt of at least one COVID-19 mRNA vaccine (BNT162b2 or mRNA-1273) in pregnancy and adverse maternal and neonatal outcomes, Kiely et al utilized a Quebec hospitalization discharge database to identify all singleton pregnancies in Quebec, Canada resulting in a live birth or stillbirth at ≥20 weeks of gestation between May 1, 2021 to June 30, 2023. The cohort was linked to four other publicly available databases. Poisson regression models were used to estimate adjusted risk ratios (aRRs) for several maternal and neonatal adverse events, and a weighted Cox proportional regression model with a time varying exposure variable was used to estimate adjusted hazard ratios (aHRs) for stillbirth. Among 140,073 singleton pregnancies resulting in live birth or stillbirth, 43.8% received at least one dose of an mRNA COVID-19 vaccine during pregnancy. Vaccination during pregnancy was not associated with an increased risk of chorioamnionitis, postpartum hemorrhage, very preterm birth or stillbirth. In fact, receipt of a COVID-19 vaccine during pregnancy was associated with a reduced risk of several maternal and neonatal adverse events, including maternal ICU admission, NICU admission, SGA and preterm birth. The approach was thoughtful, the writing is clear, and the statistical methods are appropriate and very well-executed. Overall, this is an important and well-done paper. I have very few edits, as per below:

Introduction

1) I had to look up the Canadian Immunization Research Network, as it was not immediately clear to me from the paper how the CIRN referenced in the title related to the paper. I recommend adding one to two sentences in the Introduction to explain the CIRN for those of us not based in Canada.

2) Line 52: it should be noted that "COVID-19 infection" is technically incorrect, as COVID-19 is the disease or clinical syndrome caused by SARS-CoV-2 infection. Please correct this throughout the paper and in the tables.

3) Lines 82: please remove the hyphen from the last line of the paragraph "and-maternal outcomes in Quebec".

Methods

4) Lines 133 - 136: the description of the equation used for date of conception was somewhat confusing. Please revise this to make it more clear.

5) Lines 202 - 206: can you please comment further on this decision?

Results, Subgroup and sensitivity analyses

6) Lines 271 - 275: you cite an adjusted HR of 1.23, 95% CI 1.05 - 1.43. However, table S3 lists an unadjusted HR of 1.20 (1.03 - 1.39) and adjusted HR of 1.44 (1.22 - 1.71) for only one dose during pregnancy, and very preterm birth. Please ensure consistency across the text and tables.

7) Table 1: "to" should replace "a" in the Estimated Date of Conception

Discussion

8) Line 325: please specify that the association was only observed with a single vaccine dose, not vaccination in general (which you go on to explain further in the Discussion, but should be framed in this line).

9) Lines 329 - 333: can you please clarify whether the "unexposed" group in this sensitivity analysis included only those unvaccinated in pregnancy (therefore could have been vaccinated during the prenatal period) or never received a vaccine dose? Since this subgroup included those without documented SARS-CoV-2 infection, this might also contribute to the interpretation of this somewhat surprising finding.

Overall, this is an outstanding paper. The authors should be proud. Thank you for the invitation to review your work.

VERSION 1 - AUTHOR RESPONSE

Reviewers' comments

Reviewer 1

Comment no 1: Can the authors please confirm whether all hospitals in Quebec were included in this study?

Response: Yes, all public hospitals providing general and specialized care are included in the data source used to identify the study population. We have added the following information in the manuscript to address this comment: *"It contains data on hospital stays occurring in all public hospitals providing general and specialized care"* (see lines 93-94 from the tracked-changes version, page 4)

Comment no 2: Can the authors please comment on whether the final analysed cohort can be considered to be representative?

Response: As mentioned in response to the comment no 1, we used the Quebec hospitalization discharge database to identify the cohort. All publics hospitals providing general and specialized care are included in this database and about 97% of birth in Quebec occur in hospital. Accordingly, we are confident that our cohort is representative of women who gave birth during the study period. We have added this information in the *Discussion*: *"The use of the provincial hospitalization discharge database linked with population-based vaccination registry ensures the cohort to be representative and provides accurate information on COVID-19 vaccination exposure"* (see lines 313-317 from the tracked-changes manuscript, page 10)

Comment no 3: The Tables show missing data (with some variables up to 7.4% missing). Can the authors please clarify how this missing data was handled in the analysis? Can the authors please comment on whether the missing data can be considered to be missing at random?

Response: There were 7.0% (n=9865) of missing data for material and social deprivation indices and less than 5% for infant sex (n=3802, 2.7%), pre-pregnancy medical condition (n=4817, 3.4%) and

geographical areas (n=757, 0.5%). Missing data were not assumed to be missing at random and were handled by assigning individuals with missing data to a separate (missing) category for these variables. For deprivation indices and pre-pregnancy medical condition, missingness may have occurred in individuals with an invalid health insurance number or postal codes during the study period and could also be linked to immigration status. As we did not have information on some covariates potentially associated with missingness (e.g. immigration), missing values were not assumed to be missing at random. However, the observed characteristics of individuals with and without missing data were generally similar. This, together with the relatively low proportion of missing data, suggest that the potential bias due to missingness may be low.

We have added this information in the manuscript to address this comment: *"There were 7.0% (n=9865) of missing data for material and social deprivation indices and less than 5% for infant sex (n=3802, 2.7%), pre-pregnancy medical condition (n=4817, 3.4%) and geographical areas (n=757, 0.5%). Missing data were not assumed to be missing at random and were handled by assigning individuals with missing data to a separate (missing) category for these variables"* (see lines 177 to 181 from the tracked-changes manuscript, page 7).

Comment no 4: Can the authors please consider assessing and adjusting for lockdowns and responses to the pandemic at different stages as a potential confounder? For instance, both vaccination and caesarean rates may be influenced by pandemic timeline/restrictions in place?

Response: We agree with the reviewer that lockdowns and responses to the pandemic could represent potential confounders. This issue was addressed in our analysis by including a covariate for the month and year of conception to account for different stages of the pandemic. We can also confirm that the proportion of caesarean deliveries remained constant over time. No change was made on the manuscript.

Comment no 5: Can the propensity model please be shown in full in the supplementary material?

Response: We have added details about the propensity model in the supplement (Table S3)

Comment no 6: Whilst the authors note in the study limitation that 'we did not have information on some known risk factors for the outcomes of interest, such as parity, education level, ethnicity and smoking, which could be associated with vaccination', is information on maternal BMI available?

Response: We did not have information on prepregnancy BMI specifically, but we did have data on obesity (BMI ≥ 30), which was included in the propensity score (Table S3). We have added a clarification to the definition of obesity (i.e. a BMI ≥ 30 kg/m^2) among the list of covariates: *"The following variables were considered potential confounders for the association between vaccination and maternal and neonatal outcomes: maternal age at delivery (categorized), infant sex, pre-pregnancy medical conditions (cardiovascular diseases, diabetes, hypertension, hypothyroidism, immunosuppression, obesity (BMI ≥ 30 kg/m^2) and respiratory diseases), health care worker status, tetanus-diphtheria-acellular pertussis (Tdap) vaccination during pregnancy (proxy for health behaviors), history of at least one positive SARS-CoV-2 test during pregnancy, geographical areas, month and year of conception and area-level social and material deprivation indices*". (see line 152 from the tracked-changes manuscript, page 5).

Comment no 7: Can unweighted and weighted characteristics, and the change in standardised differences between vaccination groups, please be shown in the same table (i.e. Table 1)?

Response: We have included both unweighted and weighted characteristics, along with the change in standardised differences between groups in the Table 1, as suggested by the reviewer.

Comment no 8: Thanks for providing a copy of the Research Protocol, which aligns with the methodology reported in the main article.

Response: We have added the research protocol as a supplementary file.

Reviewer 2:

Comment no 1: I had to look up the Canadian Immunization Research Network, as it was not immediately clear to me from the paper how the CIRN referenced in the title related to the paper. I recommend adding one to two sentences in the Introduction to explain the CIRN for those of us not based in Canada.

Response: CIRN is the funder for this project. We have added this information in the funding statement*: "CIRN is a national network, supported through a Canadian Institutes of Health Research (CIHR) grant, composed of key vaccine researchers who develop, and test methodologies related to the evaluation of vaccines as they pertain to safety, immunogenicity and effectiveness and program implementation and evaluation".*

Comment no 2: Line 52: it should be noted that "COVID-19 infection" is technically incorrect, as COVID-19 is the disease or clinical syndrome caused by SARS-CoV-2 infection. Please correct this throughout the paper and in the tables.

Response: We have corrected the information in the Table 1 to mention "History of SARS-CoV-2 infection during pregnancy" instead of "History of COVID-19 infection during pregnancy".

Comment no 3: Lines 82: please remove the hyphen from the last line of the paragraph "and-maternal outcomes in Quebec".

Response: The hyphen has been removed (Line 75 from the tracked-changes version (page 3))

Comment no 4: Lines 133 - 136: the description of the equation used for date of conception was somewhat confusing. Please revise this to make it more clear.

Response: We have revised the manuscript to address this comment: *"The date of conception was estimated by adding 14 days to the last menstrual period date, which was derived from the gestational age at birth (validated by ultrasound) and the date of birth available in the hospitalization records"* (see lines 124 to 127 from the tracked-changes version, page 5)

Comment no 5: Lines 202 - 206: can you please comment further on this decision?

Response: In sensitivity analyses, we limited the unexposed group to those vaccinated during the preconception or during the postpartum period, but not during pregnancy. As already mentioned in the manuscript, baseline characteristics could differ between pregnant individuals vaccinated during pregnancy and those who were never vaccinated. We then compared individuals vaccinated during pregnancy with those vaccinated before and after pregnancy, but not during. The latter group was expected to be more similar to those vaccinated during pregnancy. No change was made on the manuscript.

Comment no 6: Lines 271 - 275: you cite an adjusted HR of 1.23, 95% CI 1.05 - 1.43. However, table S3 lists an unadjusted HR of 1.20 (1.03 - 1.39) and adjusted HR of 1.44 (1.22 - 1.71) for only one dose during pregnancy, and very preterm birth. Please ensure consistency across the text and tables.

Response: The HR of 1.23, 95% CI 1.05-1.43 for very preterm birth refers to the sensitivity analysis using the Cox regression model and is presented in the last row of the Table S4. To avoid confusion, we have added this clarification in the manuscript: *"Finally, we found a significant increased risk associated with COVID-19 vaccination for very preterm birth, using a Cox regression model and considering vaccination during pregnancy as a time-varying exposure (adjusted HR 1.23, 95% CI 1.05-1.43 in the Cox model for very premature birth in Table S4 vs. adjusted RR 1.04, 95% CI 0.89-1.21 in Table 4a)"* (see lines 257-258 from the tracked-changes version, page 8).

Comment no 7: Table 1: "to" should replace "a" in the Estimated Date of Conception

Response: We have replaced "a" by "to" in the Table 1 for the estimated date of conception.

Comment no 8: Line 325: please specify that the association was only observed with a single vaccine dose, not vaccination in general (which you go on to explain further in the Discussion, but should be framed in this line).

Response: The results you are referring to pertain to exposure to at least one dose during pregnancy. We found an increased risk of very preterm birth in the sensitivity analysis using a Cox regression model. Results regarding a single vaccine dose are discussed in lines 329-333 of the submitted manuscript (lines 307-310 from the tracked-changes manuscript). To avoid confusion, we have added this clarification in the manuscript: *"The positive association with vaccination (at least one dose) observed in our sensitivity analysis for very preterm birth should be interpreted with caution since it may reflect a type 1 error due to multiple testing or residual confounding by unmeasured characteristics and should be reassessed in future studies"*(See lines 303-306 from the tracked-changes version, page 9).

Comment no 9: Lines 329 - 333: can you please clarify whether the "unexposed" group in this sensitivity analysis included only those unvaccinated in pregnancy (therefore could have been vaccinated during the prenatal period) or never received a vaccine dose? Since this subgroup included those without documented SARS-CoV-2 infection, this might also contribute to the interpretation of this somewhat surprising finding.

Response: The unexposed group was the same as the one used in the main analyses and included individuals who were unvaccinated during pregnancy. This group could include those who received a dose in preconception but none during pregnancy. As explained in the manuscript, our main hypothesis for this finding was that individuals who gave birth prematurely may have been more likely to receive only one dose during pregnancy. In addition, analyses excluding individuals with SARS-CoV-2 infection were consistent with the main results. No change was made on the manuscript.

VERSION 2 - REVIEW

**Reviewer**	**1**
**Name**	**Kelson, Zoe**

Affiliation University of Exeter, Mathematics

Date 25-Nov-2025

COI

Many thanks to the authors for their responses, clarifications, and revisions. I have no further comments.
